# Highly Specific Protein Identification by Immunoprecipitation–Mass Spectrometry Using Antifouling Microbeads

**DOI:** 10.1021/acsami.1c22734

**Published:** 2022-05-10

**Authors:** Esther van Andel, Mark Roosjen, Stef van der Zanden, Stefanie C. Lange, Dolf Weijers, Maarten M. J. Smulders, Huub F. J. Savelkoul, Han Zuilhof, Edwin J. Tijhaar

**Affiliations:** † Laboratory of Organic Chemistry, Wageningen University, Stippeneng 4, 6708 WE Wageningen, The Netherlands; ‡ Cell Biology and Immunology group, Wageningen University, De Elst 1, 6709 PG Wageningen, The Netherlands; § Laboratory of Biochemistry, Wageningen University, Stippeneng 4, 6708 WE Wageningen, The Netherlands; ∥ School of Pharmaceutical Sciences and Technology, Tianjin University, 92 Weijin Road, Tianjin 300072, People’s Republic of China; ⊥ Department of Chemical and Materials Engineering, Faculty of Engineering, King Abdulaziz University, 21589 Jeddah, Saudi Arabia

**Keywords:** antifouling, microbeads, proteomics, immunoprecipitation, mass spectrometry, zwitterionic polymer brushes, click chemistry, antibody functionalization

## Abstract

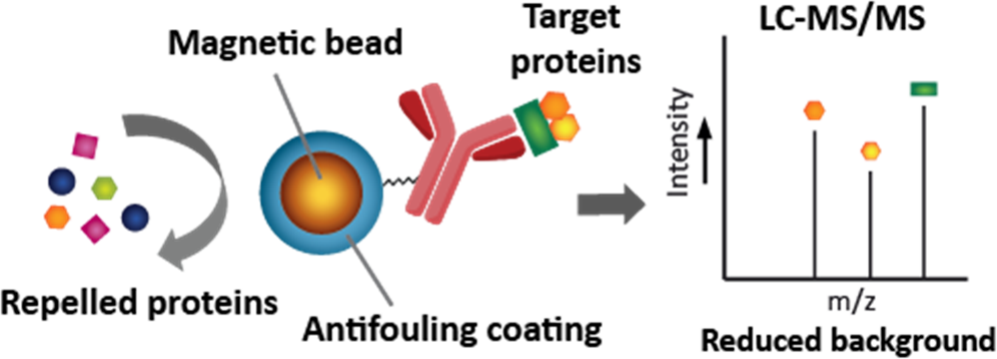

A common method to study protein complexes is immunoprecipitation (IP), followed by mass spectrometry (thus labeled: IP-MS). IP-MS has been shown to be a powerful tool to identify protein–protein interactions. It is, however, often challenging to discriminate true protein interactors from contaminating ones. Here, we describe the preparation of antifouling azide-functionalized polymer-coated beads that can be equipped with an antibody of choice via click chemistry. We show the preparation of generic immunoprecipitation beads that target the green fluorescent protein (GFP) and show how they can be used in IP-MS experiments targeting two different GFP-fusion proteins. Our antifouling beads were able to efficiently identify relevant protein–protein interactions but with a strong reduction in unwanted nonspecific protein binding compared to commercial anti-GFP beads.

## Introduction

Proteins are the workhorses of life as they take part in essentially all biological processes. In these processes, they rarely work alone, but typically act in multiprotein complexes.^1^ To fully understand biological mechanisms, both in health and disease, it is therefore crucial to reliably identify protein interaction partners.^2,3^ In the last decades, along with other techniques like the yeast two-hybrid system,^4,5^ immunoprecipitation followed by mass spectrometry (IP-MS) has emerged as a powerful tool to identify these protein–protein interactions, mainly due to the continuing improvement in sensitivity and speed of mass spectrometers.^6,7^ In a typical IP-MS experiment, a solid support with antibodies directed against a “bait” protein is used to precipitate protein complexes. The captured proteins are subsequently digested into peptides that are analyzed by mass spectrometry.^8^ The stable core subunits from a multiprotein complex are usually readily identified, but it remains challenging to distinguish subunits that, for example, bind substoichiometrically or with low affinity, from that of contaminating proteins that are nonspecifically retrieved from the biological sample.^7,9^ In fact, the majority of proteins typically identified during an IP-MS experiment are nonspecific binders.^7^ The nonspecific binders most frequently originate from proteins sticking to the solid support itself, *e.g*., the sepharose, agarose, or (hydrophilic) polymer-coated magnetic beads, and to a smaller degree to the nonspecific binding of proteins to the antibodies that are attached to the bead or to protein tags, such as green fluorescent protein (GFP).^7,10^

Several approaches have been used to discriminate true protein–protein interactions from background noise. Stringent washing conditions are easily implemented, but these do often lead to the loss of proteins that are weakly or transiently associated with the protein complex of interest.^11^ A common strategy is to use stable isotype labeling with amino acids in cell culture (SILAC),^12,13^ in which cells containing bait proteins are grown in “heavy” medium containing amino acids that are isotopically labeled, while negative control cells (without bait proteins) are grown in standard “light” medium. After the IP-MS experiment is performed, one can then discriminate a true interactor from a nonspecific binder based on the ratios between the heavy and light peaks of the identified protein. Recently, also label-free quantitative methodologies have been developed that are less laborious and more suitable for high-throughput screenings than SILAC.^9^ Moreover, software tools have also been developed and lists of common contaminants are being compiled to assist in the data analysis and interpretation of IP-MS experiments.^11,14,15^ Nonetheless, the majority of proteins identified in an IP-MS experiment are still nonspecific binders and specific interactions cannot always be unambiguously determined, especially those close to the threshold level at which signal-to-noise ratios are low.^7,9^ The above-described methodologies all seem to take nonspecific binding of proteins to the solid support as an inevitable aspect of an IP-MS experiment, thus refraining from tackling the issue at its core by reducing nonspecific binding on the solid support.

Nonspecific binding of biomolecules to solid surfaces, often referred to as fouling, is a recurring problem in many biomedical and bioanalytical applications.^16,17^ To prevent or reduce fouling, various types of antifouling surface coatings have been widely investigated, yet mainly on flat surfaces.^18−20^ Poly(ethylene glycol) (PEG)-based materials are the most frequently used and studied coatings; however, their stability and performance in real-life biological fluids like blood serum or plasma are too limited for many biomedical applications.^21−23^ Polymeric zwitterionic coatings have emerged as excellent alternatives.^16,18,24^ Their outstanding antifouling properties have been attributed to the formation of an electrostatically induced hydration layer, which facilitates the repellence of proteins.^25^ Given their success, zwitterionic coatings are increasingly implemented in biomedical applications, for example, on indwelling medical devices to reduce wear and fouling,^26,27^ to enhance the sensitivity of biosensing platforms,^28−30^ and for the production of antimicrobial surfaces.^31,32^ The identification of protein–protein interactions via IP-MS procedures relies on the ability to discriminate true interactors from nonspecific binders. We therefore anticipated that the incorporation of antifouling materials into the existing IP-MS methodologies would be highly valuable.

**Scheme 1:**
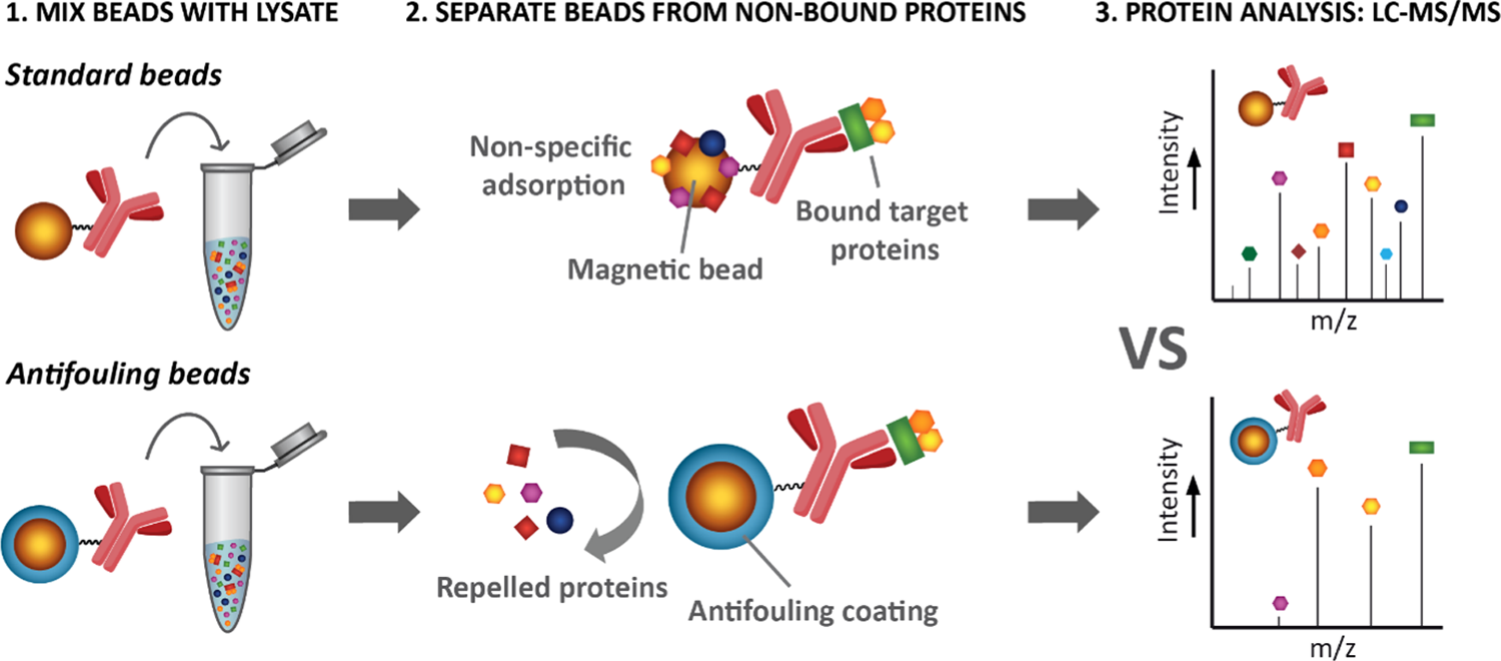
Schematic Representation of IP-MS Workflow Using Antibody-Functionalized Magnetic Beads, With or Without Antifouling Coating (1) The beads are mixed with a complex protein mixture (*e.g.*, cell lysate). (2) Beads are separated from the protein sample using a magnet. Beads with antifouling coating bind only target proteins, while other proteins are being repelled, whereas beads without antifouling coating bind target proteins but are also contaminated with nonspecifically bound proteins. (3) Subsequent protein analysis by LC-MS/MS shows a significant reduction in contaminating proteins for the antifouling beads.

As a proof of principle, we show the development of antifouling zwitterionic polymer-coated magnetic beads that can be functionalized with antibodies and then subsequently used within a standard IP-MS protocol (see Scheme 1 for a schematic representation of the IP-MS workflow). To this end, we introduced functional azide groups in the zwitterionic polymer layer of previously developed antifouling beads.^33^ These azide groups allowed for efficient attachment of antibodies via established click chemistry to these beads.^34^ To show the potential of this approach, we created generic anti-GFP beads, which we then used in IP-MS experiments targeting two different protein complexes (NuRD and PRC2^9^) from human cell lines in which one of the proteins of each complex was expressed as a GFP-fusion protein. The antifouling beads were able to specifically capture these GFP-fused bait proteins as well as the other proteins that are known to be part of the same protein complexes. The antifouling beads strongly reduced the amount of nonspecific protein binding, thereby outperforming the commercial anti-GFP beads that were used as a positive control in the IP-MS experiments.

**Figure 1:**
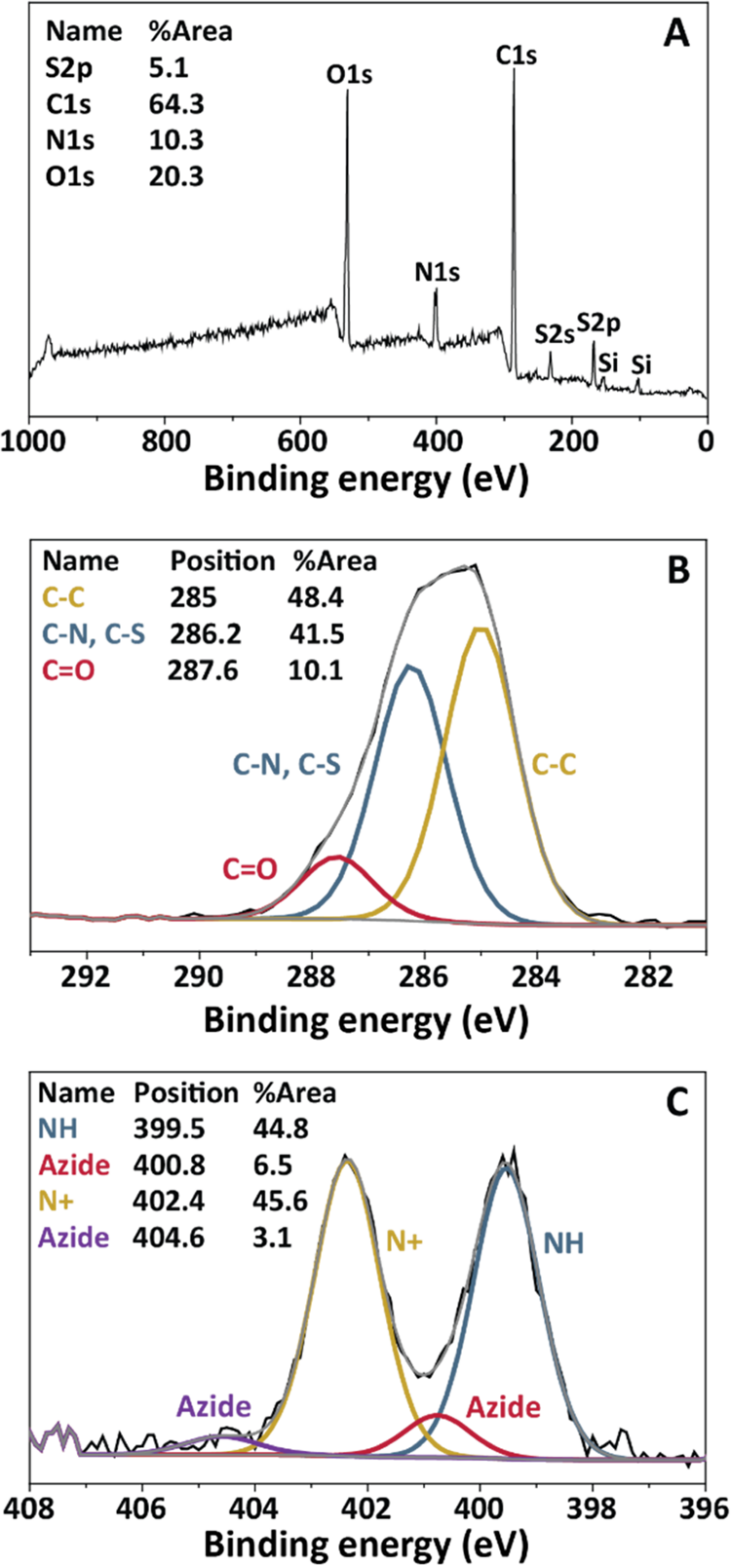
(A) XPS wide scan, (B) XPS C 1s narrow scan, and (C) XPS N 1s narrow scan of *p*SB-*co*-(azido)_8%_ beads. The spectra confirm the successful growth of zwitterionic *co*-polymer brushes of SB and azido-SB monomer from Dynabeads. The Si peaks in the wide scans can be attributed to the silicon substrate onto which the beads were drop-cast prior to XPS analysis.

## Experimental Procedures

### Materials

α-Bromoisobutyryl bromide (98%), *N*,*N*-diisopropylethylamine, copper(I) chloride (≥99%), copper(II) chloride (97%), dimethyl sulfoxide (DMSO) (anhydrous, ≥99.9%), iodoacetamide and acrylamide were purchased from Sigma-Aldrich. Dimethylformamide (DMF) for peptide synthesis (99.8%) was obtained from Acros Organics and dried over heat-activated molecular sieves (3Å), 2,2′-bipyridine (98%) was purchased from Alfa Aesar, isopropanol (HPLC grade) from BioSolve, and dichloromethane (DCM) from VWR International S.A.S.

Milli-Q water was produced with a Milli-Q Integral 3 system (Millipore). Dynabeads (Dynabeads M-270 amine; 2.8 μm diameter) were purchased from Invitrogen Life Technologies, and GFP-Trap_M (anti-GFP V_H_H coupled to magnetic microparticles) and GFP-binding protein (anti-GFP V_H_H purified protein) from Chromotek. Phycoerythrin (PE)-conjugated Goat anti-mouse IgG antibody (anti-mouse-PE, clone: Poly4053) was obtained from Biolegend. Bovine serum albumin-Alexa Fluor 488 conjugate (BSA-AF488) and EZ-Link Sulfo-NHS-LC-Biotin were obtained from Thermo Fisher, streptavidin-phycoerythrin (Strep-PE) conjugate, and streptavidin-FITC (Strep-FITC) from eBioscience. Lissamine rhodamine B PEG3 azide (Azide-Lissamine) was purchased from Tenova Pharmaceuticals, and *endo*-BCN-PEG4-NHS ester (BCN-NHS) from tebu-bio. cOmplete Protease Inhibitor Cocktail, trypsin and mouse monoclonal anti-GFP antibody (IgG_1_κ, clones 7.1 and 13.1) were obtained from Roche. Bradford reagent was purchased from Bio-Rad, fetal calf serum (FCS) from Gibco, Bolt sample buffer from Invitrogen, GelCode Blue Stain Reagent from Thermo Scientific, and goat anti-mouse IgG antibody-HRP conjugate (AP127P) from Merck.

### Synthesis

The synthesis of the 3-((3-methacrylamidopropyl)dimethylammonio)propane-1-sulfonate (**SB**) monomer was performed via an one-step procedure, and the 3-((3-azidopropyl)(3-methacrylamidopropyl)(methyl)ammonio)propane-1-sulfonate (**azido-SB**) monomer was obtained after a five-step synthesis route, both were prepared as previously described.^34^

### Bead Handling

For all collection and washing steps, beads were separated from solvent and reactants using a magnetic stand (Promega). In all cases, unless stated otherwise, reactions with beads were performed in 2 mL Eppendorf tubes; this allowed for better bead collection when using the magnetic stand. To ensure similar amounts of beads across experiments, beads were counted using a Bürker counting chamber prior to antibody immobilization, flow cytometry analysis, and IP-MS.

### Initiator Attachment

The required amount of Dynabeads (here 500 μL of bead suspension as supplied by the manufacturer, which roughly corresponds to 1 × 10^9^ beads/mL) was transferred to a glass tube with screw-cap connection. The beads were separated from the buffer by a magnet and after removal of the liquid, the beads were further dried in a vacuum oven at 50 °C for 2–4 h. The beads were resuspended in 2 mL of dry DCM, followed by the addition of 0.5 mL of *N*,*N*-diisopropylethylamine and 0.6 mL of α-bromoisobutyryl bromide to the bead suspension. The reaction tube was wrapped with aluminum foil and placed overnight on an end-over-end shaker at room temperature (RT). Afterward, the beads were washed with copious amounts of DCM, washed twice with isopropanol, and subsequently twice with Milli-Q water. This protocol was adapted from previous work.^34^

### Surface-Initiated Polymerization

Surface-initiated atom-transfer radical polymerization (ATRP) was performed as previously described but adapted to the use of beads and **azido-SB** monomer.^33,34^ All steps were performed under argon atmosphere in Schlenk flasks and solutions were transferred via argon-flushed needles. A mixture of isopropanol/Milli-Q water (20/80) was degassed by 5 min sonication and 30 min of argon bubbling. Within a glovebox, 78.1 mg (0.50 mmol) of 2,2′-bipyridine and 23.0 mg (0.23 mmol) of an Cu(I)Cl/Cu(II)Cl_2_ (9/1) mixture were added to a Schlenk flask. The flask was then transferred to the fume hood where 8.2 mL of the degassed isopropanol/water mixture was added. The resulting mixture was stirred for 15 min at RT. Meanwhile, the **azido-SB** monomer solubilized in Milli-Q water (28.9 mg, 80.0 μmol, for *p*SB-*co*-(azido)_8%_ beads) was transferred to a Schlenk flask. Milli-Q water from this aliquot was removed under reduced pressure before the **SB** monomer (269 mg, 0.920 mmol, for *p*SB-*co*-(azido)_8%_ beads) was added. To the monomer mixture, 900 μL of the brown copper/bipyridyl-containing solution was added and stirred for 15 min at RT to fully solubilize the monomers. The initiator-functionalized Dynabeads were resuspended in 200 μL of isopropanol/Milli-Q mixture and bubbled with argon for 10 min. The monomer containing ATRP solution was transferred to the beads, the flask was closed, covered with aluminum foil, and placed on a shaker at 80 rpm for 15 min at RT. The reaction was stopped by opening the sample to air, pouring the solution into an Erlenmeyer flask, and adding Milli-Q water while swirling, until the solution turned blue (which indicates the inactivation of the copper catalyst and typically takes ∼5 s). The *p*SB-*co*-(azido)_8%_-coated beads were collected using a magnet and then washed once with isopropanol/Milli-Q (1/4), twice with Milli-Q water, and twice with PBS pH 7.4. The beads were stored in PBS at 4 °C until further use.

### Bead Characterization

#### X-ray Photoelectron Spectroscopy (XPS)

XPS samples were prepared as previously described.^33^ In short, beads (in Milli-Q) were concentrated and drop-cast onto a piece of Si(111) (Siltronix, N-type, phosphorus-doped), which was cleaned by sonicating for 5 min in semiconductor-grade acetone followed by oxygen plasma treatment (Diener electronic, Femto A) for 5 min at 50% power. The samples were subsequently dried in a vacuum oven (15 mbar) at 50 °C for at least 2 h. XPS spectra were obtained using a JPS-9200 photoelectron spectrometer (JEOL, Japan) with monochromatic Al Kα X-ray radiation at 12 kV and 20 mA. The obtained spectra were analyzed using CASA XPS software (version 2.3.16 PR 1.6).

#### Dynamic Light Scattering (DLS)

Particle size measurements were performed using a Zetasizer Nano-ZS apparatus (Malvern Panalytical) equipped with a He Ne laser operating at 633 nm. Bead suspensions were prepared in ultrapure Milli-Q water in disposable cuvettes (PS 2.5 mL, CAT No. 7590, BRAND). Solutions were prepared with ca. 5–10 × 10^6^ beads in 4 mL of Milli-Q water; this concentration of beads minimized settling of the beads on the bottom of the tubes during the length of the DLS experiments. Measurements were performed at 25 °C. The Zetasizer Malvern version 7.02 software was used to acquire the data. Measurements were performed using a 2 min equilibration time and 10–100 runs (automatically determined) per measurement.

### Antibody Production and Purification

Monoclonal TA99 antibody (IgG_2a_) was produced by a hybridoma cell line obtained from the American Tissue Culture Collection (ATCC HB-8704) that was cultured in Roswell Park Memorial Institute (RPMI) medium (Lonza), supplemented with l-glutamine, 10% fetal calf serum (FCS), 100 μg/mL penicillin, and 100 μg/mL streptomycin, at 37 °C and 5% CO_2_. The antibody was isolated from the hybridoma cell line supernatant by repetitive passing of the supernatant over a Pierce Thiophilic Adsorption column (Thermo Fisher Scientific) according to the manufacturer’s protocol, followed by elution of the antibody with PBS. The concentration of the antibody was determined by a NanoDrop 1000 spectrophotometer and checked for its purity using sodium dodecyl sulfate–polyacrylamide gel electrophoresis (SDS-PAGE).

### Antibody Functionalization

#### BCN-NHS

The antibody of choice (the mouse IgG_2a_ TA99 or camelid V_H_H anti-GFP antibody) was concentrated and transferred to PBS (pH 6.5) using Amicon Ultra 0.5 mL, 0.3 kDa molecular weight cutoff (MWCO) centrifugal filter tubes (Merck Millipore) according to the manufacturer’s instructions. The antibodies were subsequently labeled at a 4 mg/mL antibody concentration with an *endo*-BCN-PEG4-NHS ester (BCN-NHS) linker in a 1:8 ratio (assuming that the molecular mass of the mouse IgG TA99 is 150 kDa and that of aGFP is 13.9 kDa) by adding the BCN-NHS linker from a stock solution in dry DMF, to a final DMF concentration of 10%. The antibody was incubated with the linker at RT for 1 h. The reaction was stopped by removing unreacted BCN-NHS by washing three times with 500 μL of PBS pH 6.5 using the Amicon Ultra (0.3 kDa MWCO) filter tubes (in which the first two times 10 min centrifugation at 2100*g* was used and the third time 30 min centrifugation at 2100*g*).

#### Azide-Lissamine Staining

BCN-labeled antibodies (4 mg/mL) were incubated with Azide-Lissamine in PBS pH 7.4 with an antibody/Azide-Lissamine molar ratio of 1:20 and a final DMSO concentration of 10%. The reaction was carried out overnight under ambient conditions at RT, with the reaction mixture protected from light. The resulting reaction mixture was directly used for SDS-PAGE without further purification.

### Antibody Attachment to Beads

BCN-labeled antibodies were attached to *p*SB-*co*-(azido)_8%_ beads (∼50 × 10^6^ beads) in PBS pH 7.4, in a final volume of 50 μL in a PCR tube. For the TA99 antibody either 0.5, 1, 2, 4 or 8 mg/L antibody was used, for the aGFP antibody 0.25, 1 or 4 mg/mL. The tube was fixed on an end-over-end shaker, which was placed vertically, and incubated overnight at RT. To obtain homogeneous attachment it was crucial to have a proper dispersion of the beads; for further details on this, see section ‘Ensuring Sample Homogeneity’. The beads were transferred to a 2 mL Eppendorf tube and washed three times with PBS pH 7.4. The beads were stored in PBS at 4 °C.

### Serum Biotinylation

Bovine serum was obtained and biotinylated as previously described.^33^ In short, sera of three adult cows were pooled and heated at 56 °C for 30 min (to inactivate complement proteins). Serum proteins were biotinylated using an EZ-Link Sulfo-NHS-LC-Biotin reagent, using the manufacturer’s instructions. Assuming that the average molecular weight of serum proteins is 70 kDa, 50 equivalents of sulfo-NHS-biotin to serum proteins was used. The reaction was carried out at RT for 60 min. Nonbound reagents were removed using a desalting PD-10 column (Sephadex, from GE Healthcare), following the manufacturer’s gravity protocol with PBS as eluent. The concentration of the obtained biotinylated serum (serum-biotin) was adjusted to 10% serum solution (∼6 mg/mL serum proteins) with PBS. Bovine blood sample collection was approved by the Board on Animal Ethics and Experiments from Wageningen University (DEC number: 2014005.b).

### Flow Cytometry

TA99-functionalized *p*SB-*co*-(azido)_8%_ beads (∼2 × 10^6^ beads) were incubated in PBS, BSA-AF488 (0.5 mg/mL) or anti-mouse-PE (1:50 dilution). Serum binding was evaluated by incubating the beads with serum-biotin (10% solution) followed by Strep-FITC (1:200 dilution). Specific binding of anti-mouse-PE was evaluated by incubating the beads with a mixture of anti-mouse-PE and BSA-AF488, or by first incubating the beads with serum-biotin followed by incubation with a mixture of anti-mouse-PE and Strep-FITC. All protein solutions were diluted in PBS.

Anti-GFP-functionalized *p*SB-co-(azido)_8%_ beads and Chromotek bead (2 × 10^6^ beads per sample) were incubated in PBS or free GFP (10 μg/mL in PBS) to evaluate GFP-binding capacity, and with serum-biotin (10%) followed by Strep-PE (1:50 dilution) to evaluate the antifouling ability. To measure both GFP capture and antifouling from the same solution, the beads were incubated in a mixture of GFP and serum-biotin, followed by staining with Strep-PE. All incubation steps were performed for 30 min in 100 μL of total volume on an end-over-end shaker at RT and protected from light using aluminum foil, followed by washing three times with 1 mL of PBS. The beads were subsequently resuspended in 500 μL of PBS and transferred to a FACS tube.

All samples with beads were analyzed with a BD FACS Canto A (BD Biosciences) flow cytometer. For each sample, 10,000 beads were measured. GFP, BSA-AF488, and Strep-FITC were visualized using the FITC channel, and fouling was visualized by Strep-PE using the PE channel. Data analysis was performed using FlowJo LLC Software V10.

### Ensuring Sample Homogeneity

To obtain homogeneous samples, *i.e.*, sharp peaks by flow cytometry, it was essential to keep the beads in suspension during all steps as the beads settle quickly. In the first step, in which amine-terminated Dynabeads were reacted with α-bromoisobutyryl bromide, this was achieved by placing the reaction tube on an end-over-end shaker. During ATRP, the homogeneity of the samples was maintained by shaking on an incubator shaker at 80 rpm. We used a wide flask (diameter of ∼3.5 cm) with a round-shaped bottom under an angle of approximately 45° to create a large surface area (if the tube that is used is too narrow the beads will settle despite the shaking). All functionalization, staining, and protein incubation steps were performed in separate PCR tubes which were fixed on an end-over-end shaker (4 rpm) of which the rotating wheel was placed exactly perpendicular to the table. If the rotating wheel is placed under an angle the beads will settle against the wall of the tube, which should be avoided. The smaller the volume the more challenging it becomes to keep all beads in suspension. With small volumes it works well to use narrow PCR tubes as this prevents the solution to “roll” through the tubes, avoiding beads getting stuck in the lid and not being mixed. Volumes ranging from 20–150 μL can be reliably used when using the PCR tubes. When larger volumes are desired, 2 mL Eppendorf tubes can be reliably used with volumes ≥200 μL. In this case, the exact placement of the tube on the end-over-end shaker is less crucial as the entire bead volume will be “rolling” through the tube (for this reason 1.5 mL Eppendorf tubes together with small volumes are less suitable as part of the sample will be retained at the bottom of the tube and part is likely to get stuck in the lid, leading to inhomogeneous samples). We used a maximum of 2 × 10^6^ beads per μL of sample. It is also recommended to quickly collect all beads after each incubation step by a pulse spin of a few seconds in an Eppendorf centrifuge.

### Cells

#### Cell Culture

Wild-type (WT) HeLa cells (HeLa B-50 from ATCC (CRL-12401)) and HeLa cells stably expressing MBD3-GFP or EED-GFP (kindly provided by Prof. Dr. M. Vermeulen from the Radboud Institute of Molecular Life Sciences (RIMLS))^9^ were grown in Dulbecco’s modified Eagle’s medium (DMEM, Gibco) supplemented with 10% FCS, 100 μg/mL penicillin, and 100 μg/mL streptomycin at 37 °C and 5% CO_2_.

#### Cellular Extracts

For whole-cell lysates, cells were harvested at ∼90% confluency using trypsin, washed twice with cold PBS, and centrifuged at 4 °C for 5 min at 400*g*. For whole-cell extracts of WT HeLa cells, the cells were resuspended in five pellet volumes of cold lysis buffer (150 mM NaCl, 50 mM Tris pH 8.0, 1% NP40 detergent, and 20% glycerol) and incubated for 1 h on an end-over-end shaker at 4 °C. The cell lysates were centrifuged in Eppendorf tubes for 20 min at 21,000*g* (maximum speed) and 4 °C. The supernatants were stored at −80 °C.

Nuclear extracts of WT and MBD3-GFP or EED-GFP HeLa cells were prepared according to Smits et al.^9^ The cells were resuspended in five volumes of cold Buffer A (10 mM HEPES/KOH pH 7.9, 1.5 mM MgCl_2_, 10 mM KCl) and incubated on ice for 10 min in a 15 mL tube. The cells were centrifuged for 5 min at 400*g* and resuspended in two cell volumes of Buffer A, supplemented with 0.15% NP40 and complete protease inhibitor. The cells were transferred to a Dounce homogenizer and after 30–40 strokes with a Type B pestle, the resulting lysate was centrifuged for 15 min at 3200*g* at 4 °C. The pellet, containing the nuclei, was washed once with 1 mL of PBS and centrifuged for 5 min at 3900*g* at 4 °C. The pellet was resuspended in two pellet volumes of nuclei lysis buffer (420 mM NaCl, 20 mM HEPES/KOH pH 7.9, 20% v/v glycerol, 2 mM MgCl_2_, 0.2 mM EDTA, 0.1% NP40, 0.5 mM DTT and complete protease inhibitors) and transferred to an Eppendorf tube. The suspension was incubated for 1 h on an end-over-end shaker at 4 °C and centrifuged for 30 min at 18,000*g* at 4 °C. The supernatants were aliquoted and stored at −80 °C until further use. Protein concentrations were determined using Bradford reagent (Bio-Rad).

### Immunoprecipitations

For the immunoprecipitations targeting MBD3-GFP ∼18.6 million beads were used, which is equal to 10 μL of Chromotek bead slurry, whereas for the EED-GFP experiments, ∼93 million beads were used, which is equal to 50 μL of Chromotek bead slurry. All measurements were carried out in triplicates.

WT Hela whole-cell lysate was used for the evaluation of nonspecific protein binding on the beads. Nonmodified Dynabeads, *p*SB-*co*-(aGFP)_8%_ Dynabeads and Chromotek beads were equilibrated three times by magnetic separation in whole-cell lysate buffer (150 mM NaCl, 50 mM Tris pH 8.0, 20% glycerol, and 1% NP40). Next, the beads were incubated for 90 min at 4 °C with 1 mg of whole-cell lysate. Beads were subsequently washed three times in whole-cell lysis buffer, three times in PBS + 1% NP40, and three times in 50 mM NH_4_HCO_3_. After the washing steps, beads were subjected to on-bead trypsin digestion (see below).

For the *co*-IP-MS experiments, nuclear extracts of WT, MBD3-GFP, or EED-GFP expressing HeLa cells were subjected to GFP enrichment using Chromotek or *p*SB-*co*-(aGFP)_8%_ beads. In brief, beads were equilibrated three times in nuclei lysis buffer by magnetic separation at 4 °C. After equilibration, 1 mg of nuclear extract was added, and the beads were incubated for 90 min at 4 °C on an end-over-end shaker. The beads were washed twice in nuclei lysis buffer, twice in nuclei lysis buffer without NP40, and twice in 50 mM NH_4_HCO_3_. For mass spectrometry analysis, the beads were subjected to on-bead trypsin digestion (see below).

### SDS-PAGE

Antibody labeling was evaluated using Any kD Mini-PROTEAN TGX Precast Protein gels and Precision Plus Protein Dual Color Standards from Bio-Rad, with 10 μg protein per sample. Fluorescently labeled proteins were visualized using a Bio-Rad ChemiDoc XRS+ apparatus using the standard EtBr filter (580 AF 120 Band Pass Filter) followed by staining with GelCode Blue Stain Reagent to visualize all proteins.

### Sample Preparation for Mass Spectrometry

Bead-precipitated proteins were subjected to on-bead trypsin digestion as described by Smith et al.^S9^ In brief, beads were resuspended in elution buffer (2 M urea, 100 mM Tris pH 7.5, and 10 mM DTT) to partly unfold the proteins, and incubated for 20 min at 25 °C. After 20 min 50 mM acrylamide was added and the sample was further incubated for 30 min at 25 °C in the dark. After alkylation, 0.35 μg trypsin was added, followed by overnight incubation at 25 °C. After overnight trypsin digestion, the obtained peptides were desalted and concentrated by C18 Stagetips according to Rappsilber et al.,^35^ with the modification that on top of the Stagetips, 1 mg of LiChroprep C18 beads were used. After Stagetip processing, peptides were applied to online nanoLC-MS/MS using a 60 min acetonitrile gradient from 8–50% in 0.1% formic acid. Spectra were recorded on an LTQ-XL mass spectrometer (Thermo Scientific) and analyzed according to Wendrich *et al.*(36) Data visualization was performed in Adobe Illustrator and R. The data are represented as volcano plots in which the −^10^log-transformed *P*-values of a False Discovery Rate (FDR)-corrected t-test are plotted against the ^2^log of the relative label-free quantification (LFQ) intensities, in line with previously reported methods.^36^ Identified proteins are considered significant (green, red, and blue dots) with a ^2^log fold-change (FC)> 2 and a *P*-value <0.05, as indicated by the gray lines.

## Results and Discussion

### Preparation of Zwitterionic Polymer-Coated Beads with Azide Groups for Click Chemistry

**Scheme 2:**
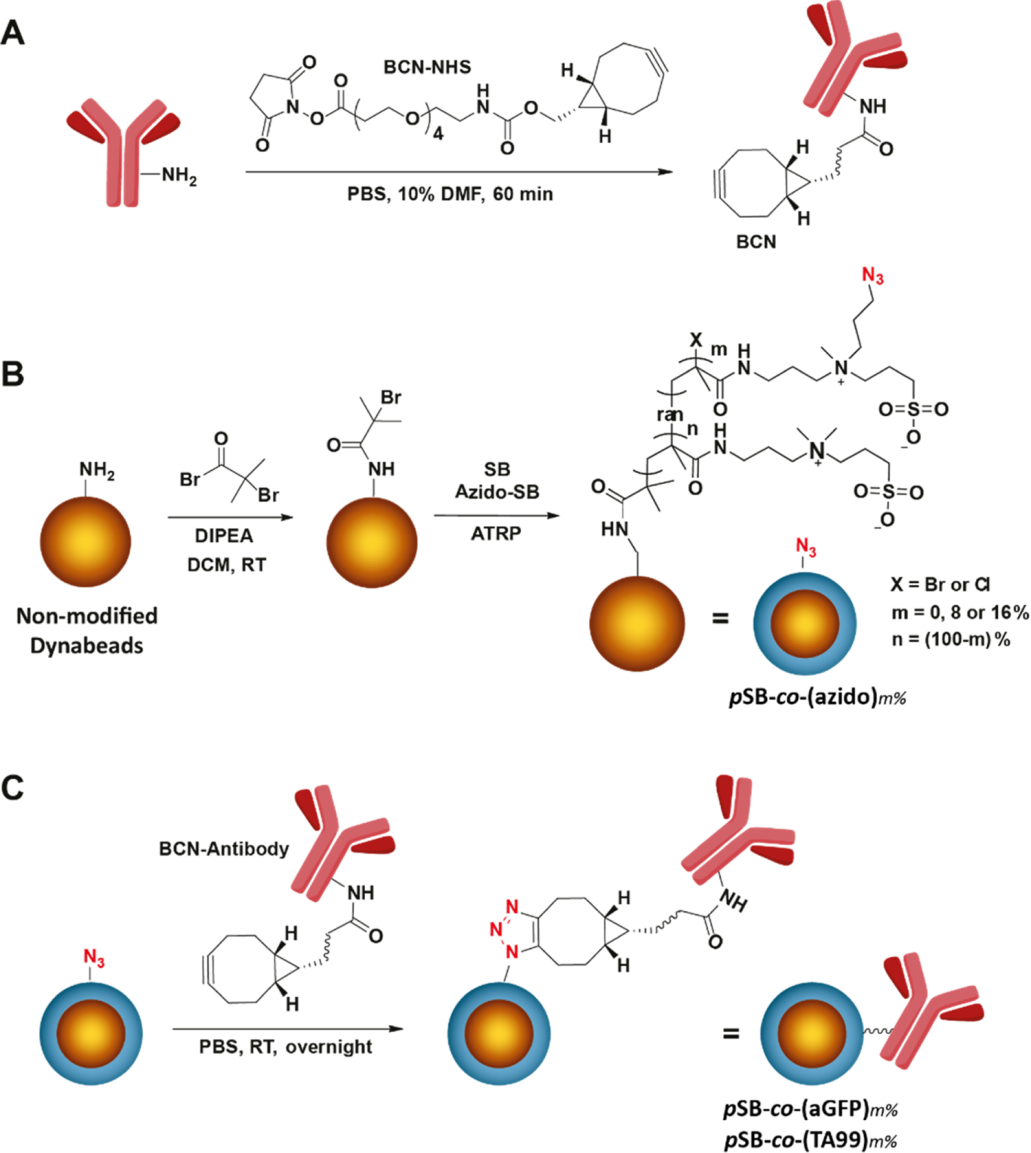
Overview of Chemical Modifications of Antibodies and Amine-Terminated Beads to Yield Magnetic Antifouling Beads with Coupled Antibodies (A) Functionalization of antibodies using endo-BCN-PEG4-NHS ester (BCN-NHS) linker. The NHS part of the linker reacts with free primary amines of the antibody to yield BCN-functionalized antibodies. (B) Installation of the ATRP initiator by reacting α-bromoisobutyryl bromide with amine-terminated Dynabeads, followed by the co-polymerization of a standard sulfobetaine (SB) methacrylamide with an azide-functionalized sulfobetaine (azido-SB) using ATRP, to generate antifouling beads with *m*% of azide moieties. (C) Combining the BCN-functionalized antibodies from (A) with the functional antifouling beads of (B) via the strain-promoted azide–alkyne cycloaddition (SPAAC) reaction of BCN with the incorporated azides.

To obtain antifouling polymer-coated microbeads we used surface-initiated atom-transfer radical polymerization (SI-ATRP),^37^ in which the zwitterionic polymer brushes are grown from the surface via controlled radical polymerization. SI-ATRP has become the method of choice to create high-performance antifouling layers, as it yields densely packed coatings of tunable thicknesses.^16,38^ We combined here our previous work on antifouling beads made by SI-ATRP^33^ with that on azide-functionalized coatings on flat surfaces^34^ (see Scheme 2B). The azide functionalities can be incorporated by the copolymerization of a standard zwitterionic sulfobetaine monomer (**SB**) with that of a tailor-made azide-functionalized sulfobetaine (**azido-SB**),^34^ enabling the incorporation of azide moieties at the desired percentages while retaining a fully zwitterionic brush.

The first step was the installation of an ATRP initiator by reacting commercially available, amine-terminated magnetic Dynabeads with bromoisobutyryl bromide. This was followed by the copolymerization of the standard **SB** monomer with **azido-SB**, with **azido-SB** percentages *m* = 0–16%, to obtain *p*SB-*co*-(azido)_*m*%_ beads. The successful growth of the copolymer brushes was first confirmed by X-ray Photoelectron Spectroscopy (XPS), a surface analysis technique often used to study polymer brushes. The XPS wide scan revealed the appearance of two sulfur peaks (168 eV for S 2p, 232 eV for S 2s) that originate from the negatively charged sulfonate group (Figure 1A, *p*SB-*co*-(azido)_8%_ beads). The XPS C 1s spectrum (Figure 1B) showed the C–C peak at 285.0 eV, the carbon atoms next to a heteroatom (C–O, C–N, and C–S) at 286.2 eV and the carbonyl peak at 287.6 eV. The experimentally derived percentages of 48.4% (C–C), 41.5% (C–O, C–N, and C–S) and 10.1% (carbonyl) fit well to the theoretical percentages of 50.0, 41.6, and 8.3%, respectively. The 1:1 ratio of the positively charged ammonium (402.4 eV) and amide (399.5 eV) peaks that is characteristic for these amide-linked zwitterionic moieties, is clearly seen in the XPS N 1s spectrum (Figure 1C), confirming polymer brushes of sufficient thickness.^33^ Moreover, from deconvolution of the N 1s spectrum of *p*SB-*co*-(azido)_8%_ beads, two additional peaks (400.8 and 404.6 eV, 2:1 ratio) can be observed that correspond to the azide functionalities of the incorporated **azido-SB** monomers. From the area of those azide peaks, it followed that 7% of **azido-SB** was incorporated within the polymer brushes, which is close to the aimed 8% **azido-SB**. The azide peaks fitted in Figure 1C are near the limit of quantification by XPS; for more pronounced azide peaks, see Figure S3, which shows the N 1s spectrum of *p*SB-*co*-(azido)_16%_ beads. In line with this, the azide signals of *p*SB-*co*-(azido)_*m*%_ beads with *m* ≤ 4% were too low to be detected in the XPS 1 Ns spectra (data not shown). The XPS wide scans and the C and N 1s spectra of *p*SB-*co*-(azido)_*m*%_ beads are virtually identical to previously shown **azido-SB** brushes on flat surfaces.^34^ In conclusion, zwitterionic antifouling microbeads were successfully obtained, with variable percentages of azide functionalities, that can be further functionalized via click chemistry.

**Figure 2:**
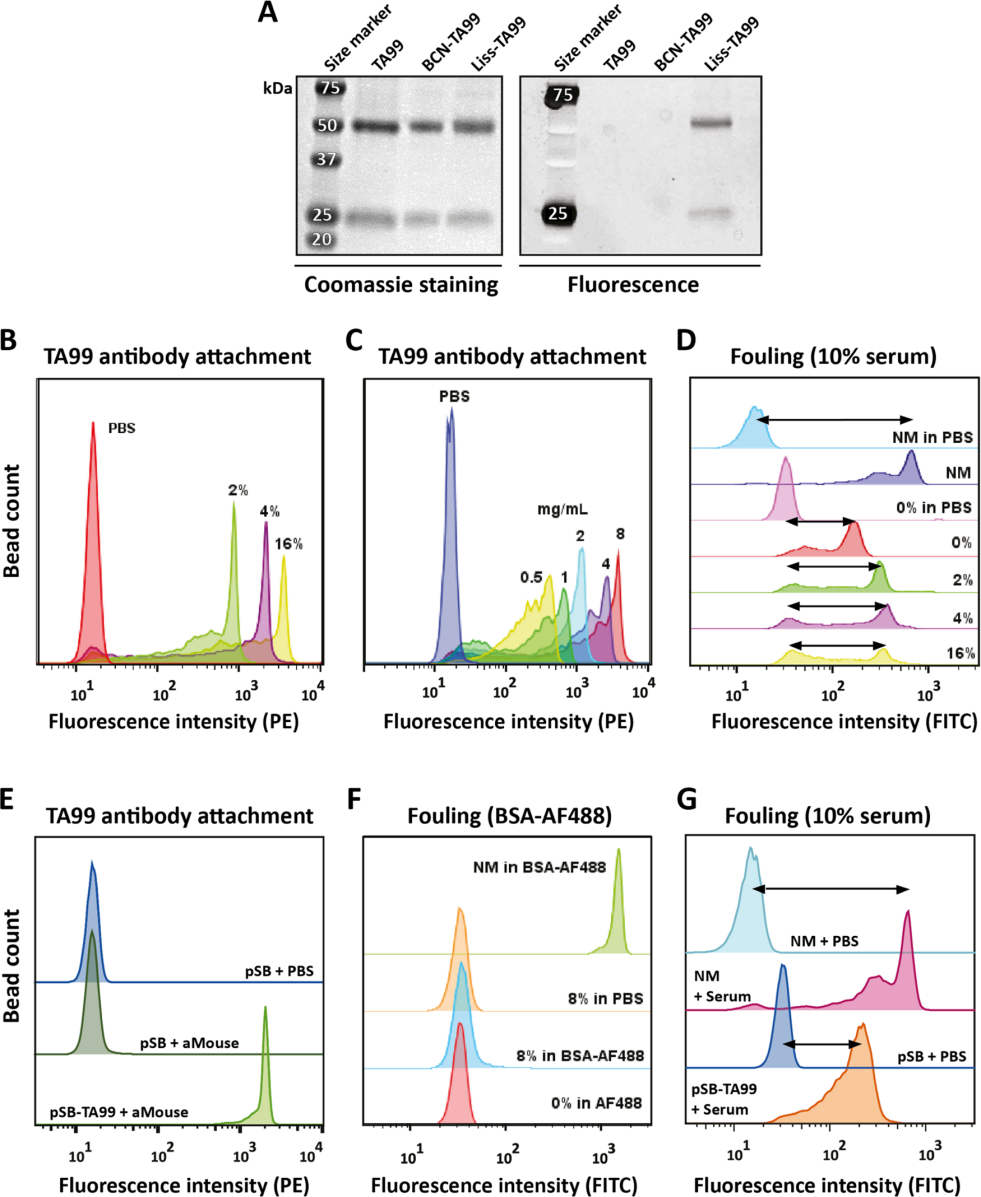
Coupling efficiency and antifouling performance of TA99 antibody to *p*SB-*co*-(azido)_*m*%_ (pSB) beads. Panels B-G display flow cytometry data. (A) Evaluation of BCN-NHS coupling to TA99 antibody by SDS-PAGE analysis. Untreated TA99 antibody, TA99 labeled with BCN-NHS (BCN-TA99), and BCN-TA99 reacted with Azide-Lissamine (Liss-TA99) were visualized by fluorescence and Coomassie blue staining. (B) *p*SB-*co*-(TA99)_*m*%_ beads with *m* = 2, 4, and 16%, TA99 attachment is shown using an anti-mouse-PE antibody. (C) *p*SB-*co*-(TA99)_8%_ beads in PBS are shown together with *p*SB-*co*-(TA99)_8%_ beads prepared using 0.5, 1, 2, 4, or 8 mg/mL TA99, TA99 was stained with an anti-mouse-PE antibody. (D) *p*SB-*co*-(azido)_8%_ (= 0% beads) and nonmodified (NM) beads in PBS, and NM and *p*SB-*co*-(TA99)_*m*%_ (*m* = 0, 2, 4, 16) beads incubated with serum-biotin followed by Strep-FITC. *p*SB-*co*-(TA99)_*m*%_ beads were prepared using a 4 mg/mL TA99 antibody concentration. (E) *p*SB-*co*-(azido)_8%_ (pSB) in PBS, and *p*SB-*co*-(azido)_8%_ and *p*SB-*co*-(TA99)_8%_ (pSB-TA99) beads stained with an anti-mouse-PE (aMouse) antibody. (F) *p*SB-*co*-(TA99)_8%_ beads in PBS and incubated with BSA-AF488, as well as pSB and nonmodified (NM) beads incubated with BSA-AF488. Broad histogram peaks are most likely a result of inhomogeneous sample mixing, see the Experimental Procedure section.

Next, the polymer-coated beads were analyzed by dynamic light scattering (DLS) to evaluate the polymer brush thickness in water. Figure S4 shows the hydrodynamic diameter of nonmodified beads versus polymer-coated beads, with *p*SB-*co*-(azido)_8%_ beads as an example. The nonmodified beads were measured to have a diameter of 2877 ± 62 nm, which corresponds well to the size of 2.8 μm as provided by the supplier. The *p*SB-*co*-(azido)_8%_ beads were measured to have an average diameter of 3439 ± 112 nm, which corresponds to an average polymer thickness of about 280 nm. Sulfobetaine-based polymer brushes are known for their excellent hydration properties and have been shown to be able to swell up to a factor of 2.3–2.5 in thickness (and even more with high ionic strength).^39,40^ This suggests a dry polymer brush thickness over 100 nm, which, together with the 1:1 ratio of the ammonium versus amide peak by XPS, confirms the successful growth of a zwitterion polymer brush layer of sufficient thickness.

### Optimization of Antibody Attachment to Antifouling Beads via Click Chemistry

The introduction of azide moieties within the antifouling coating allows for the functionalization of the polymer brushes via click chemistry, *e.g*., by the copper-catalyzed azide–alkyne cycloaddition (CuAAC)^41^ or strain-promoted azide–alkyne cycloaddition (SPAAC).^42^ We have previously shown the incorporation of mannose and biotin on zwitterionic sulfobetaine polymer-coated beads via CuAAC and SPAAC, respectively.^33,34^ To use the antifouling beads for immunoprecipitations, the beads need to be functionalized with antibodies. A commercially available BCN-NHS linker was chosen for this, enabling coupling of antibodies to the *p*SB-*co*-(azido)_*m*%_ beads (see Scheme 2). The primary amines of the antibody can react with the NHS group to form a stable covalent amide bond, while the BCN moiety can react with the azides within the polymer layer via the aforementioned SPAAC reaction,^42^ to form a stable triazole-containing link. Antibodies were first reacted with the less stable NHS part of the BCN-NHS linker, followed by reaction of the BCN-antibody conjugate with the *p*SB-*co*-(azido)_*m*%_ beads. Performing the reactions in this order allowed for the removal of unreacted or hydrolyzed BCN-NHS linker from the samples.

The optimal conditions to attach an antibody to the *p*SB-*co*-(azido)_*m*%_ beads were first evaluated using a model mouse IgG antibody (we chose an anti-TRP1 clone TA99 monoclonal mouse antibody that we had available in large quantities). The successful attachment of the aforementioned BCN-NHS linker to the TA99 antibody was verified by labeling the BCN-TA99 antibodies with an azide-functionalized fluorophore (Azide-Lissamine). Unmodified TA99, BCN-TA99, and Lissamine-TA99 were loaded on an SDS-PAGE gel, after which a fluorescence image was taken to visualize the Lissamine, followed by Coomassie blue staining (visualizing all proteins). The Coomassie-stained gel showed clear bands corresponding to the heavy (∼50 kDa) and the light chains (∼25 kDa) of the TA99 antibody in all samples (see Figure 2A). The fluorescence image only showed the heavy and light chain of Lissamine-TA99, confirming the successful reaction of BCN-NHS with TA99 and the subsequent reaction of BCN-NHS with the Azide-Lissamine. The BCN-TA99 antibodies were then used to prepare a variety of *p*SB-*co*-(TA99)_*m*%_ beads. The highest loading of antibody on the beads that could be achieved without introducing a significant amount of fouling on the same beads, was considered as the optimum. The percentages of **azido-SB** that were used are *m* = 0, 2, 4, 8 and 16%, and the antibody concentration during the coupling reaction to the beads 0.5, 1, 2, 4 and 8 mg/mL. The antibody attachment to the antifouling beads and the amount of fouling after antibody attachment were evaluated by flow cytometry (see Figure 2B–E). We previously established a method to study specific protein binding as well as (anti)fouling on micron-sized beads by flow cytometry, using a variety of fluorescently labeled proteins.^33,43,44^ Here we used similar procedures. A significant advantage of flow cytometry is the ability to analyze millions of beads within a single experiment. Besides that, evaluation of the antifouling beads by flow cytometry allows for analysis of the exact same beads that can later be used for IP-MS experiments.

Successful attachment of the TA99 antibody was evaluated by incubating with a commercially available anti-mouse antibody that is fluorescently labeled with phycoerythrin (PE). A clear shift in PE-fluorescence was seen for *p*SB-*co*-(TA99)_*m*%_ beads compared to beads in PBS (see Figure 2B,C), demonstrating successful attachment of the TA99 antibody onto the polymer-coated beads. Staining *p*SB-*co*-(azido)_*m*%_ beads with the anti-mouse antibody did not result in any change in fluorescence intensity, excluding the possibility of nonspecific binding of the anti-mouse antibody (see Figure 2E for an example with *m* = 8%). Figure 2B depicts *p*SB-*co*-(TA99)_*m*%_ beads with 2, 4, and 16% **azido**-**SB**; the more **azido-SB** was incorporated in the polymer brushes, the more TA99 was immobilized. The concentration of TA99 during the immobilization step is also of influence on the amount of TA99 on the beads, as can be seen in Figure 2C. The higher the concentration, the higher the amount of immobilized TA99.

The antifouling performance of the beads was first examined by incubating the beads with fluorescently labeled bovine serum albumin (BSA-AF488). The median fluorescent intensity (MFI) of the beads was used as a measure for the extent of protein fouling. Figure 2F shows an example of *p*SB-*co*-(TA99)_8%_ beads prepared with 4 mg/mL of TA99; the TA99 antibody-functionalized beads did not show any increase in fluorescence, and thus no increase in fouling, when incubated with this single-protein BSA solution. Next, *p*SB-*co*-(TA99)_*m*%_ beads were also incubated in a 10% solution of biotinylated serum (serum-biotin). This serum solution has a 12 times higher total protein concentration than the single-protein BSA solution, and also contains a range of other proteins. Fouling of the beads with biotinylated serum proteins was detected with fluorescently labeled streptavidin (Strep-FITC). The *p*SB-*co*-(TA99)_*m*%_ beads showed an increase in fluorescence after incubation and staining of this serum-biotin solution (see Figure 2D), and the amount of fouling increases with higher amounts of immobilized TA99. However, the amount of fouling was in all cases much less than that for nonmodified Dynabeads (NM). Despite the attachment of an antibody, the antifouling properties of *p*SB-*co*-(azido)_*m*%_ beads could largely be maintained. It should be noted that within these initial optimization experiments, a relatively large fluorescence distribution can be observed for most samples (histogram plot showing broad peaks). When working with small volumes, it is challenging to keep the beads well-dispersed during all incubation times. Alongside the first experiments, we developed strategies to maximize bead homogeneity, see “Ensuring sample homogeneity” of the Experimental Procedures section. From the performed pilot experiments, we selected *p*SB-*co*-(azido)_8%_ beads combined with antibody coupling at a concentration of 4 mg/mL as the optimal balance between high antibody loading, limited amount of fouling, and appropriate usage of valuable in-house obtained materials (**azido-SB**, *p*SB-*co*-(azido)_8%_, and BCN-TA99). The attachment of TA99 to *p*SB-*co*-(azido)_8%_ beads, and the amount of fouling on those beads were confirmed with another experiment (see Figure 2E–G). Clear TA99 attachment and a limited amount of fouling (factor 4 less based on MFI compared to nonmodified beads) were again established. We then used the same strategy to attach an anti-GFP antibody to the zwitterionic antifouling beads (see below).

### Selective Capture of GFP by Generic Antifouling Anti-GFP Microbeads

GFP-fusion proteins are widely used to study protein–protein interactions.^12,45^ Using standard molecular cloning techniques, a genetic fusion between a gene of interest and the gene encoding GFP can be readily obtained and subsequently expressed in cells or whole organisms.^45,46^ The GFP-fusion proteins may then be used for multiple purposes including the visualization of dynamic cellular events and immunoprecipitations.^9,47^ When utilized for immunoprecipitations, beads coupled to GFP-specific antibodies are often used to separate the GFP-fusion protein from other cellular proteins. Proteins that interact with such a GFP-protein-of-interest fusion will be *co*-purified and can subsequently be identified by mass spectrometry. GFP itself has no major contribution to the *co*-purification of contaminating proteins.^12^ For this kind of IP-MS experiments, Chromotek GFP-Trap magnetic beads are widely used.^9^ The anti-GFP antibody coupled to these magnetic beads is the 13.9 kDa V_H_H domain (GFP-binding domain, also called GFP-nanobody) of a 27 kDa single-chain camelid antibody with a high affinity for GFP. We set out to generate a generic antifouling bead that can be used to pull-down GFP-fusion proteins by attaching the exact same camelid V_H_H anti-GFP antibody (aGFP) to *p*SB-*co*-(azido)_8%_ beads, enabling a fair comparison between these beads and the Chromotek GFP-Trap beads. The significantly smaller size of the camelid antibody V_H_H fragment, compared to standard IgG antibodies of most mammals of around 150 kDa, will likely aid in limiting the amount of fouling.

**Figure 3:**
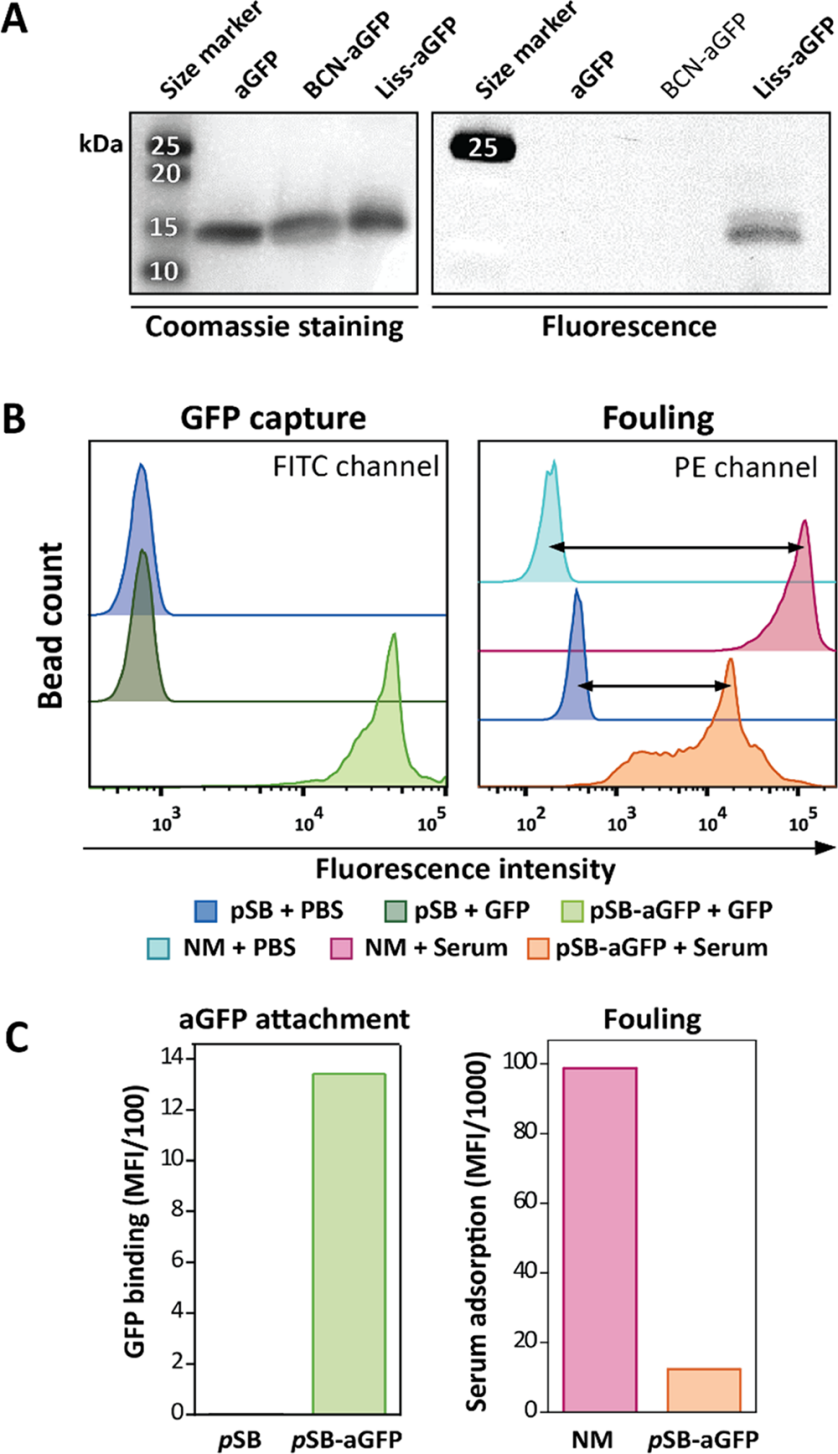
Coupling efficiency of anti-GFP camelid antibody (aGFP) to *p*SB-coated beads and antifouling performance. (A) Evaluation of BCN-NHS coupling to aGFP by SDS-PAGE analysis. Untreated aGFP antibody, aGFP labeled with BCN-NHS (BCN-aGFP), and BCN-aGFP reacted with Azide-Lissamine (Liss-aGFP) were visualized by Coomassie blue staining and fluorescence. (B) Flow cytometry data of *p*SB-*co*-(azido)_8%_ (*p*SB) beads, *p*SB-*co*-(aGFP)_8%_ (*p*SB-aGFP) beads prepared using a 4 mg/mL aGFP concentration, and nonmodified Dynabeads (NM). aGFP antibody attachment is shown by capturing free GFP (FITC channel) and fouling is visualized by incubating with 10% serum-biotin followed by staining with Strep-PE (PE channel). (C) Bar plots summarizing flow cytometry data of (B) using the median fluorescent intensities. Bead MFI values were corrected for their auto-fluorescence by subtracting MFI values of beads incubated in PBS.

As the first step, aGFP was labeled with BCN-NHS at a 1:8 antibody to linker ratio to obtain BCN-aGFP (see Scheme 2A for a schematic representation of the reaction). The attachment of the BCN linker to the antibody fragment was evaluated, in the same way as for the TA99 antibody, by reacting the BCN-aGFP conjugate with Azide-Lissamine. The Coomassie-stained gel showed in all lanes clear bands at the expected size of 13.9 kDa that corresponds to the aGFP antibody (V_H_H domain only). The fluorescence image showed a fluorescent band as a result of the presence of the Lissamine-aGFP, indicating the successful labeling of aGFP by BCN-NHS. The BCN-aGFP antibody was then coupled to *p*SB-*co*-(azido)_8%_ beads, in a similar way as described for the TA99 antibody, to obtain *p*SB-*co*-(aGFP)_8%_ beads and those beads were then analyzed by flow cytometry. Per sample, 10,000 beads were analyzed, the histogram plots as well as the median fluorescent intensity of the beads displayed as a bar plot are depicted in (Figure 3B,C). The attachment of functional BCN-aGFP to the beads was confirmed by capturing free GFP spiked in PBS (Figure 3B). There is no detectable fluorescence signal when antifouling *p*SB-*co*-(azido)_8%_ beads (*i.e*., beads without the antibody) were incubated with free GFP in PBS, demonstrating that GFP does not adsorb to the beads in a nonspecific manner. With the anti-GFP-coupled *p*SB-*co*-(aGFP)_8%_ beads however, there is a clear fluorescence signal, demonstrating selective capture of GFP. The amount of fouling observed here with the pSB-*co*-(aGFP)_8%_ beads in biotinylated serum is lower compared to the pilot experiments with the TA99 mouse IgG antibody (respectively, 8 versus 4 times lower compared to nonmodified beads), and this is likely attributed to the much smaller size of the 13.9 kDa anti-GFP camelid antibody fragment compared to the mouse IgG antibody with a MW of around 150 kDa.

**Figure 4:**
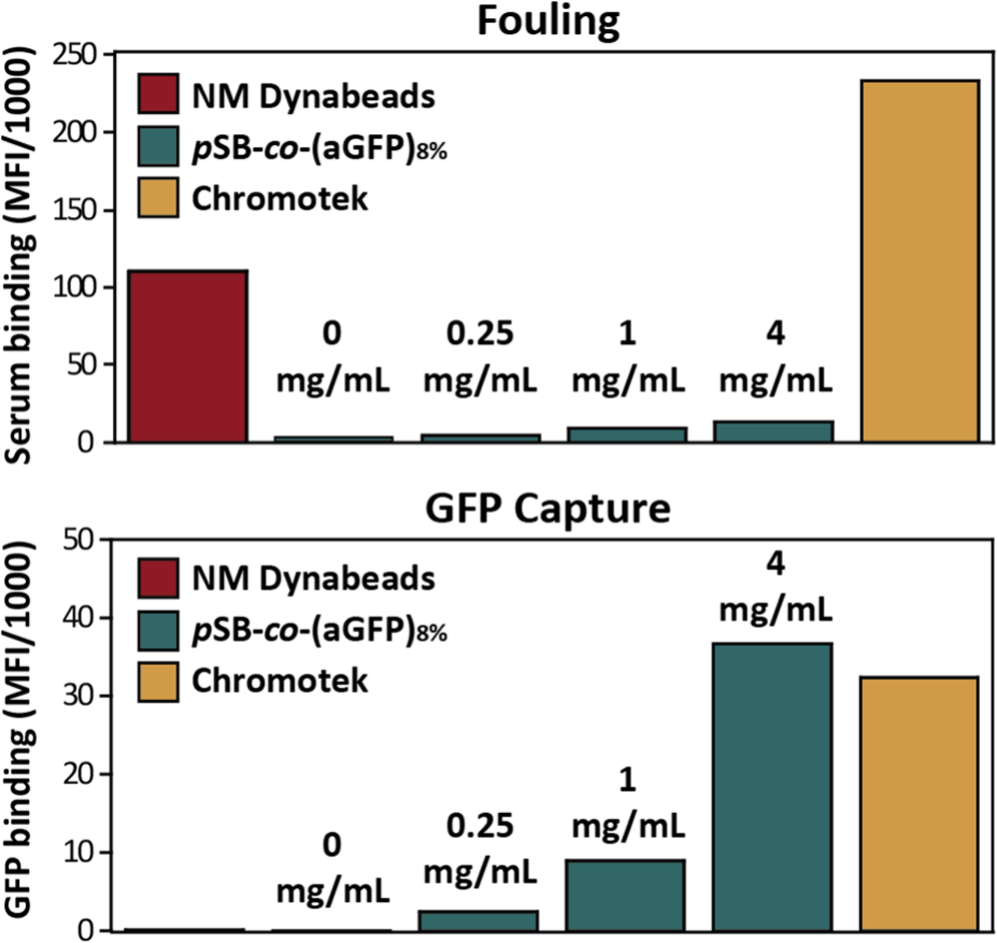
Flow cytometry data showing fouling and GFP capture by aGFP Chromotek beads, nonmodified Dynabeads, and *p*SB-*co*-(aGFP)_8%_ beads prepared with an aGFP antibody concentration of 0, 0.25, 1, or 4 mg/mL. Note that the 0 mg/mL samples are identical to the *p*SB-*co*-(azido)_8%_ beads. All beads were incubated in 10% biotinylated serum spiked with GFP (10 μg/mL), and after washing incubated with Strep-PE to stain for fouling by biotinylated serum proteins. Median fluorescence intensities (MFI) of all samples were corrected by subtracting the MFI values of their corresponding beads incubated with PBS only.

Here and in previous work,^33^ we have established that the *p*SB-*co*-(azido)_*m*%_ beads prevent or reduce fouling significantly compared to corresponding nonmodified beads. To benchmark the performance of *p*SB-*co*-(aGFP)_8%_ beads, both regarding GFP-binding efficiency and antifouling properties, a comparison was made with the widely used commercially available magnetic Chromotek GFP-Trap beads. The Chromotek beads were compared with the *p*SB-*co*-(azido)_8%_ (the 0 mg/mL samples in Figure 4; *i.e., p*SB-coated beads without aGFP attached), and *p*SB-*co*-(aGFP)_8%_ beads that were prepared at a BCN-aGFP concentration of 0.25, 1, or 4 mg/mL. All beads were incubated with 10% biotinylated serum solution, which was spiked with free GFP, followed by subsequent staining with Strep-PE to detect unspecific binding of biotinylated serum proteins.

For each type of bead, fouling and GFP capture were measured simultaneously (in two different channels) by flow cytometry. Figure 4 summarizes the intensities of 10,000 beads per sample (see Figure S9 for histogram plots). All *p*SB-*co*-(aGFP)_8%_ beads show a substantial reduction in fouling compared to nonmodified Dynabeads, and a slight increase in fouling with an increasing amount of attached aGFP antibody. Chromotek beads showed even more fouling than nonmodified Dynabeads. When comparing the amount of captured GFP, there is negligible nonspecific binding of GFP to nonmodified Dynabeads and *p*SB-*co*-(azido)_8%_ beads (the 0 mg/mL sample), but clear GFP binding by all three *p*SB-*co*-(aGFP)_8%_ beads in an almost linear relationship to the amount of coupled aGFP. The amount of captured GFP protein by Chromotek beads was most comparable to *p*SB-*co*-(aGFP)_8%_ beads prepared with 4 mg/mL aGFP. Comparability in GFP-binding capacity is of most importance when comparing the ability to pull-down protein(s) of interest. In the subsequent immunoprecipitation experiments, we therefore used the 4 mg/mL *p*SB-*co*-(aGFP)_8%_ beads for comparison with the Chromotek beads.

In Figure 4 (aGFP) and Figure 2D (TA99), it was observed that coupling of an increased amount of antibody to the beads, leads to increased fouling on those beads. In other words, when using a highly efficient antifouling coating the extent of fouling is largely determined by the amount of attached antibody. The amount of antibody coupled to the beads can be varied in three ways: (a) by changing the percentage of incorporated **azido-SB** (higher percentages leads to more antibody attachment, see Figure 2B); (b) by varying the antibody concentration during the coupling of the BCN-antibody to the azide-bearing beads (higher concentration leads to more antibody attachment, see Figures 2C and 4); and (c) by changing the BCN-NHS to antibody ratio. Depending on the application, the balance between, on the one hand, high antibody loading and corresponding binding capacity, and on the other hand, the degree of fouling, might differ and thus should be optimized accordingly.

### Selective Capture of GFP-Fusion Proteins by IP-MS Using Antifouling Microbeads

Since the *p*SB-*co*-(aGFP)_8%_ beads were able to capture similar amounts of GFP as Chromotek GFP-Trap beads (Figure 4)—but with much less nonspecific binding—it was anticipated that the *p*SB-*co*-(aGFP)_8%_ beads had sufficient antibody loading for their use in immunoprecipitations in which GFP-fusion proteins are captured from cellular extracts.

We performed two types of IP experiments, one using a Methyl-CpG Binding Domain Protein 3 (MBD3) GFP-fusion protein and one using an embryonic ectoderm development (EED) GFP-fusion protein. The MBD3 protein binds to hydroxymethylated DNA and assembles in the nucleosome remodeling and deacetylase (NuRD) complex.^48^ The EED protein is known to function in the polycomb repressive complex 2 (PRC2), which is a regulator of epigenetic states via the methylation of histone H3.^49^ The MBD3/NuRD and EED/PRC2 complexes have both been well characterized^48−51^ and Chromotek beads have been previously used to target these proteins in immunoprecipitation experiments,^9^ making them good candidates to test the performance of our new *p*SB-*co*-(aGFP)_8%_ beads. Both the MBD3-GFP and EED-GFP gene fusions were created using bacterial artificial chromosome (BAC) recombineering to ensure near endogenous expression to avoid artifacts associated with overexpression.^46,52^ Both fusion proteins were stably expressed in a HeLa cell line.

**Figure 5:**
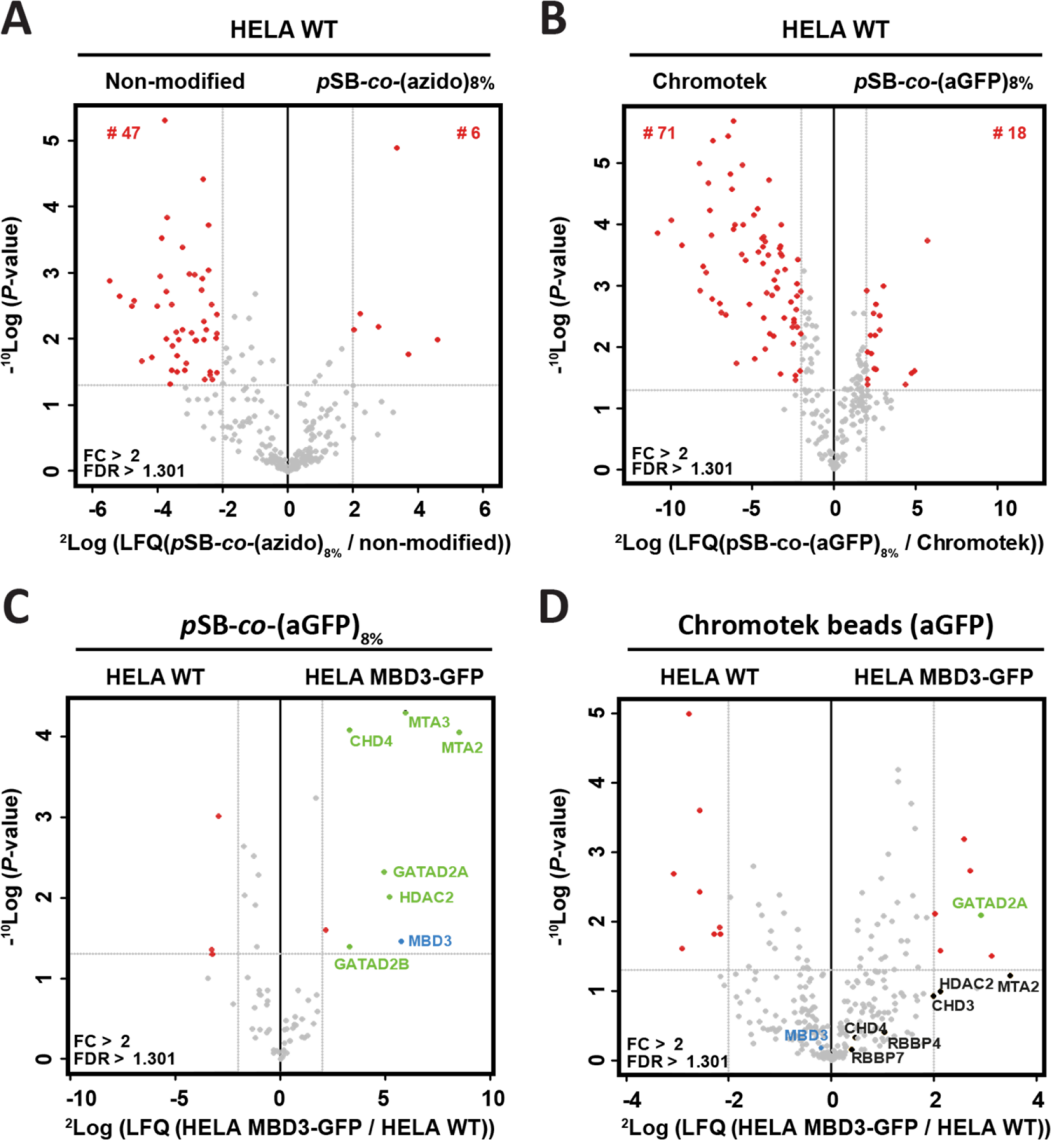
Volcano plots of mass spectrometry analysis of protein enrichment by immunoprecipitation, using nonmodified, *p*SB-*co*-(azido)_8%_, *p*SB-*co*-(aGFP)_8%_, and Chromotek beads. For each sample, ∼18 million beads were used. Statistically enriched proteins are identified using an FDR-corrected t-test. The −^10^log-transformed *P*-values of the *t*-test (*y*-axis) are plotted against the ^2^log-transformed relative label-free quantification (LFQ) intensities (*x*-axis). Proteins are considered significant (red, blue, and green dots) with a ^2^log fold-change (FC)> 2 and a *P*-value <0.05, as indicated by gray lines. (A) Nonmodified and *p*SB-*co*-(azido)_8%_ beads subjected to WT Hela whole-cell lysate, (B) *p*SB-*co*-(aGFP)_8%_ and Chromotek bead subjected to WT HeLa whole-cell lysate, and (C) *p*SB-*co*-(aGFP)_8%_ subjected to WT HeLa nuclear extract and MBD3-GFP HeLa nuclear extract and (D) Chromotek subjected to WT HeLa nuclear extract and MBD3-GFP HeLa nuclear extract.

To evaluate the amount of nonspecific binding on nonmodified Dynabeads, *p*SB-*co*-(azido)_8%_, *p*SB-*co*-(aGFP)_8%_, and Chromotek beads in an immunoprecipitation experiment, the beads were first incubated in whole-cell lysate prepared from WT Hela cells (*i.e.*, without the MBD3-GFP or EED-GFP target protein) and analyzed by nanoLC-MS/MS. The data were visualized in volcano plots (see Figure 5A,B, and Supporting Information Figure S10) in which the statistical significance (*y*-axis) of identified proteins, represented as dots, is plotted against the relative label-free quantification intensities (LFQ) between two samples (*x*-axis), in line with previously reported methods.^9,50^ In other words, the *x*-axis shows the relative difference between the samples; the protein intensities of a first sample are related to that of a second sample and expressed as ^2^log of that ratio (*i.e.*, the fold-change (FC)). The *y-*axis, on the other hand, is a measure for statistical significance, displayed as the ^10^log of the *P-*value (the higher the value, the higher the significance). Identified proteins that have a *P*-value <0.05 and are 4 times more expressed in one sample compared to the other sample (^2^log(FC)> 2) are presented as red dots, while proteins that do not fulfill these criteria are presented as gray dots. In whole-cell lysates of WT Hela cells, 47 nonspecifically bound proteins were identified that were more abundant on nonmodified Dynabeads compared to *p*SB-*co*-(azido)_8%_ beads, whereas only six nonspecifically bound proteins were identified that were more abundant on *p*SB-*co*-(azido)_8%_ compared to nonmodified beads (Figure 5A). Likewise, 71 nonspecifically bound proteins were identified that were more abundant using Chromotek beads compared to 18 nonspecifically bound proteins with *p*SB-*co*-(aGFP)_8%_ beads. Moreover, the nonspecifically bound proteins on the Chromotek beads are more abundant (*i.e.*, higher FC on Chromotek beads) than the proteins found on the *p*SB-*co*-(aGFP)_8%_ beads. Together this shows that, also by MS/MS, the zwitterionic *p*SB-based coating greatly reduces the amount of nonspecifically bound proteins.

Having established a greatly reduced amount of fouling on *p*SB-*co*-(aGFP)_8%_ beads compared to the Chromotek beads, the beads were subjected to *co*-IP-MS using MBD3-GFP-containing HeLa extracts. In an ideal *co*-IP experiment, only the bait protein (in this case, the MBD3-GFP protein, blue dots in Figure 5C,D) and its interaction partners (green dots) are detected, as any other protein might incorrectly be identified as a binding partner of the bait protein. When using tagged/fusion proteins as bait protein for IP-MS, it is common practice to minimize the risk of false positives using a protein extract identical to that of the bait protein, but without the tagged bait protein itself, as a negative control.^9^ Only the proteins that are sufficiently enriched (*i.e.*, with a ^2^log(FC)> 2) in the protein extract expressing the tagged bait protein, are considered as valuable candidates. The same methodology was followed here: *p*SB-*co*-(aGFP)_8%_ and Chromotek beads were separately incubated in nuclear extracts of MBD3-GFP expressing HeLa cells (bait samples) as well as in nuclear extracts of WT HeLa cells (control samples). Under the conditions tested, only one relevant known interaction partner (GATAD2A) of MBD3 could be identified with the Chromotek beads (see the green dot in Figure 5D). The bait MBD3-GFP-fusion protein (blue dot) as well as some other proteins of the NuRD complex (black dots) were only visible within the background, while at the same time five proteins were incorrectly identified as relevant candidates (red dots in the top right quadrant of Figure 5D). Our *p*SB-*co*-(aGFP)_8%_ beads, on the other hand, were capable of enriching the MBD3-GFP protein (blue dot, Figure 5C) and identifying eight known interaction partners of MBD3 (green dots).^9,48,50^ Only one protein (red dot, top right quadrant of Figure 5C), close to the FC> 2 threshold, would under these criteria just falsely be identified as one of the MBD3 interaction partners. Noteworthy, the total number of detected proteins was much higher for the Chromotek beads than for the *p*SB-*co*-(aGFP)_8%_ beads, whereas the amount of meaningful identified proteins, proteins from the NuRD complex, was considerably higher for the *p*SB-*co*-(aGFP)_8%_ beads. As the number of beads, nuclear extract solutions, and the reaction conditions were identical for both types of beads, and the amount of GFP captured in flow cytometry experiments was highly similar, the difference between them is most likely attributed to their difference in fouling. The fouling proteins do not only increase the risk of incorrectly identified protein–protein interactions but also hamper the identification of true interactors (as seen here for the Chromotek beads). These results stress the importance of reducing the nonspecific binding of proteins within an IP-MS experiment.

**Figure 6:**
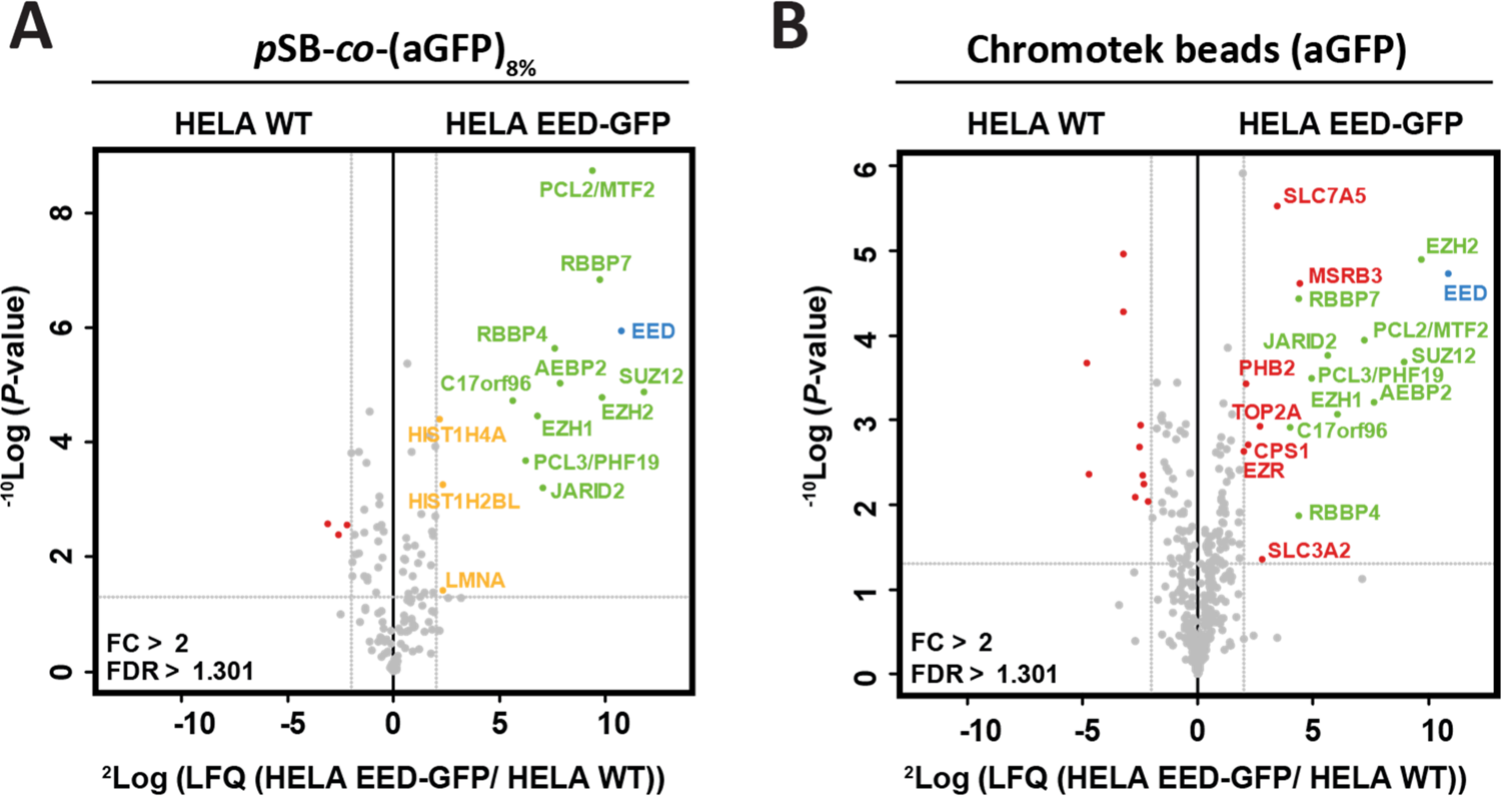
Mass spectrometry-based analysis of protein enrichment using (A) *p*SB-*co*-(aGFP)_8%_ and (B) Chromotek beads (∼93 million beads per sample), represented as volcano plots. *p*SB-*co*-(aGFP)_8%_ and Chromotek beads were incubated in either WT HeLa nuclear extract or EED-GFP HeLa nuclear extracts. Statistically enriched proteins were identified using an FDR-corrected t-test. The −^10^log-transformed *P*-values of the *t*-test (*y*-axis) are plotted against the ^2^log-transformed relative label-free quantification (LFQ) intensities (*x*-axis).

It should be noted that the IP-MS experiment using MBD3-GFP as bait protein was performed with a limited number of beads (equal to 10 μL of Chromotek slurry) while the supplier recommends a minimum of at least 25 μL slurry. This could explain the relatively poor performance of the Chromotek beads in this experiment. However, the *p*SB-*co*-(aGFP)_8%_ beads were used with an equivalent amount of beads, suggesting that more Chromotek beads are needed for a successful IP-MS experiment than with our novel beads. For the next IP-MS experiment, in which we targeted the EED-GFP protein, we therefore used for both types of beads the number of beads that equals 50 μL slurry of Chromotek beads. When looking at the total amount of captured proteins (all dots, Figure 6), it is again evident that the *p*SB-*co*-(aGFP)_8%_ beads showed less background signal. For the Chromotek beads, 378 unique proteins were identified as background signal (fouling proteins, gray dots), whereas only 118 proteins were identified for the *p*SB-*co*-(aGFP)_8%_ beads. Both the Chromotek beads as well as the *p*SB-*co*-(aGFP)_8%_ beads were able to capture the three core subunits of the PRC2 complex: EED, EZH2, and SUZ12, as well as all of the other previously identified binding partners by Smits et al. that occur at >0.1 stoichiometry relative to the EED bait protein: RBBP47, EZH1, JARID2, C17orf96, AEBP2, and PCL23 (Figure 6, green dots and labels).^9,49^ With the *p*SB-*co*-(aGFP)_8%_ beads, three additional proteins scored just positive: LMNA, HIST1H4A, and HIST1H2BL (Figure 6, yellow dots and labels). The LMNA protein is, like the PRC2 complex, a chromatin regulator,^49,53^ and was previously shown to also interact with one another.^54,55^ The RBBP4/7 proteins operate in several protein complexes and can bind to histone H4,^56^ whereas ABP2 and JARID2 can bind to histone 2.^57^ The PRC2 complex, the histone proteins and the LMNA proteins are thus found in close proximity to each other within the cell nucleus, and the LMNA, HIST1H4A, and HIST1H2BL proteins might in fact reflect true protein–protein interactors with the PRC2 complex (hence the labeling of these proteins in yellow).

The Chromotek beads in this experiment were also able to capture the core EED core subunits as well as multiple other proteins known to interact with this complex (see Figure 6B). However, the Chromotek beads also precipitated nine proteins (red dots and labels) that could not be related to the PRC2 complex and must therefore be nonspecific binders. This demonstrates again that the *p*SB-*co*-(aGFP)_8%_ beads are better in discriminating between nonspecifically bound proteins and true protein–protein interactions.

Here, we show, as a proof of principle, that antifouling beads have the ability to greatly improve the performance of an IP-MS experiment. Better identification of true protein–protein interactions can be achieved by limiting the amount of nonspecific protein binding (and hence reducing potential false-positive protein candidates), which can also improve the sensitivity of detecting true protein interactors. Depending on the intended use, the antifouling beads can be further optimized and/or tailored to the specific application and/or protein(s) of interest. Examples of further improvements are: (1) reducing the amount of fouling even further with newly developed antifouling materials, polymers made from poly[*N*-(2-hydroxypropyl) methacrylamide] show high potential,^43,58−60^ and (2) oriented antibody immobilization, which may reduce the amount of immobilized antibody that is needed.^61,62^

## Conclusions

The use of antifouling antibody-coated beads, that can be used within existing IP-MS procedures, strongly reduces the problem of contaminating proteins, resulting in both a strong reduction in false-positively identified protein–protein interactors and a more sensitive detection of true association partners. Such antifouling sulfobetaine polymer brushes were obtained by the *co*-polymerization of a standard sulfobetaine (**SB**) and a functionalizable azide-containing sulfobetaine (**azido-SB**) from the surface of microbeads, followed by the coupling of camelid anti-GFP antibody fragments (aGFP). The antifouling aGFP beads showed similar GFP-binding efficiency but significantly decreased fouling compared to commercially available aGFP beads. We hereby show the importance of reducing the nonspecific binding of proteins within IP-MS experiments and demonstrate that the incorporation of antifouling coatings has great potential in diminishing contaminating proteins, which can strongly facilitate the identification of true protein–protein interactors in newly studied complexes. The antibody-functionalized antifouling microbeads described here were used within IP-MS experiments but can be easily used for other applications, such as for protein purification or for diagnostic purposes.

## Supporting Information Available

XPS spectra, full-size SDS-PAGE gels, flow cytometry, and IP-MS data (PDF)
