# Supplementary material for: Highly Specific Protein Identification by Immunoprecipitation–Mass Spectrometry Using Antifouling Microbeads
Source: ACS Appl Mater Interfaces. 2022 May 10;14(20):23102–16. doi: 10.1021/acsami.1c22734 (PMC9136845; doi:10.1021/acsami.1c22734)
Supplement: Supplementary file 1 — am1c22734_si_001.pdf [file am1c22734_si_001.pdf]

Supporting Information

Highly specific protein identification by immunoprecipitation-mass spectrometry using antifouling microbeads

Esther van Andel,[a,b] Mark Roosjen,[c] Stef van der Zanden,[b] Stefanie C. Lange,[a] Dolf Weijers,[c] Maarten M. J. Smulders,[a] Huub F. J. Savelkoul,[b] Han Zuilhof,[a,d,e,] Edwin J. Tijhaar [b,*]*

[a] Laboratory of Organic Chemistry, Wageningen University, Stippeneng 4, 6708 WE Wageningen, The Netherlands. [b] Cell Biology and Immunology group, Wageningen University, De Elst 1, 6709 PG Wageningen, The Netherlands. [c] Laboratory of Biochemistry, Wageningen University, Stippeneng 4, 6708 WE Wageningen, The Netherlands. [d] School of Pharmaceutical Sciences and Technology, Tianjin University, 92 Weijin Road, Tianjin 300072, People's Republic of China. [e] Department of Chemical and Materials Engineering, King Abdulaziz University, Jeddah 21589, Saudi Arabia.

E-mail: Han.Zuilhof@wur.nl, Edwin.Tijhaar@wur.nl

TABLE OF CONTENTS

1. Additional XPS Spectra S-3

2. Dynamic Light Scattering data S-4

3. Full size SDS-PAGE Gels S-7

4. Additional Flow Cytometry Data S-9

5. Additional IP-MS Data S-11

1. ADDITIONAL XPS SPECTRA

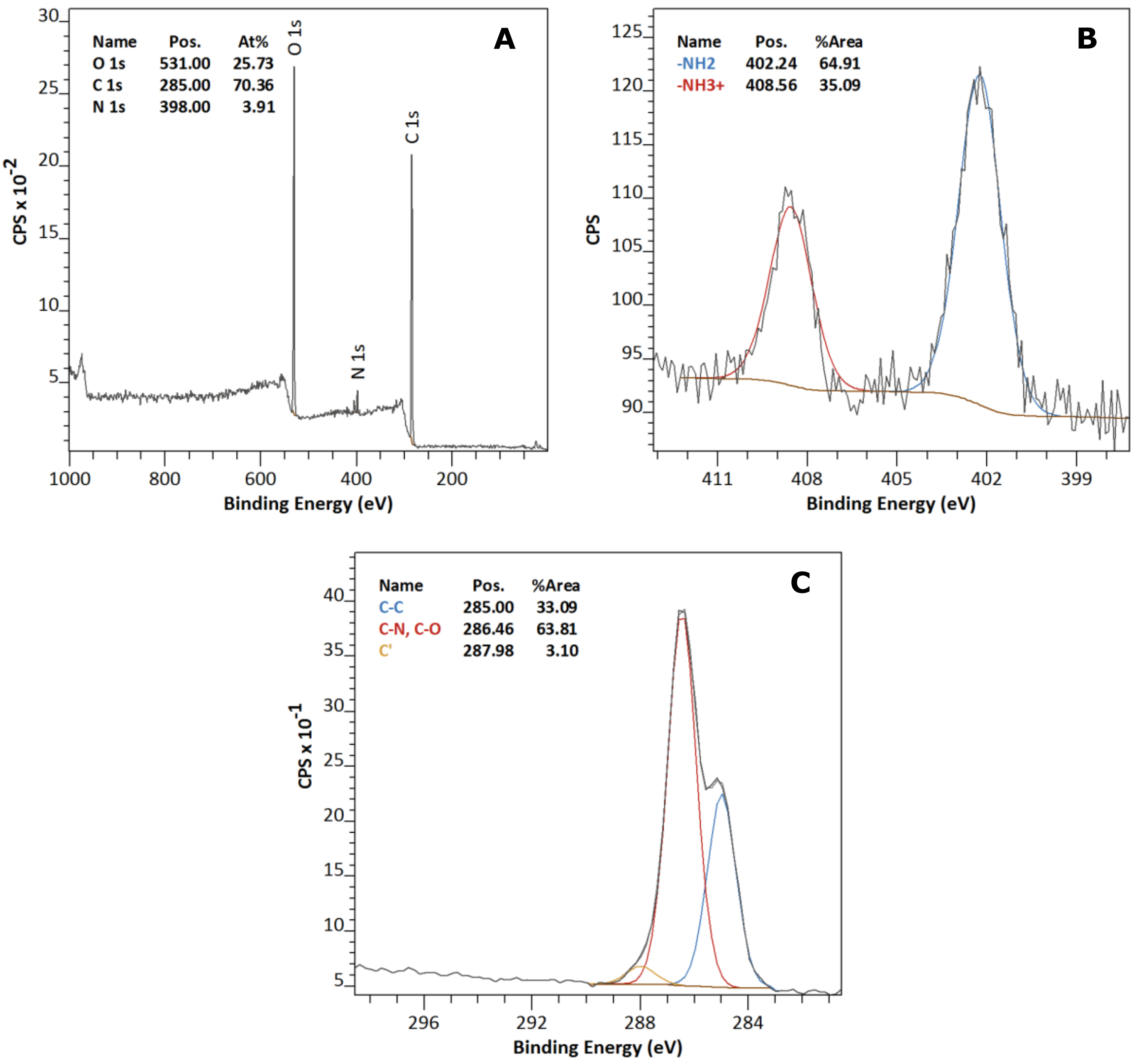

Figure S1. XPS spectra of non-modified Dynabeads (as obtained from the commercial supplier): (A) wide-scan and narrow-scans of (B) N 1s and (C) C 1s. C’ probably originates from a C–O or C–N species.

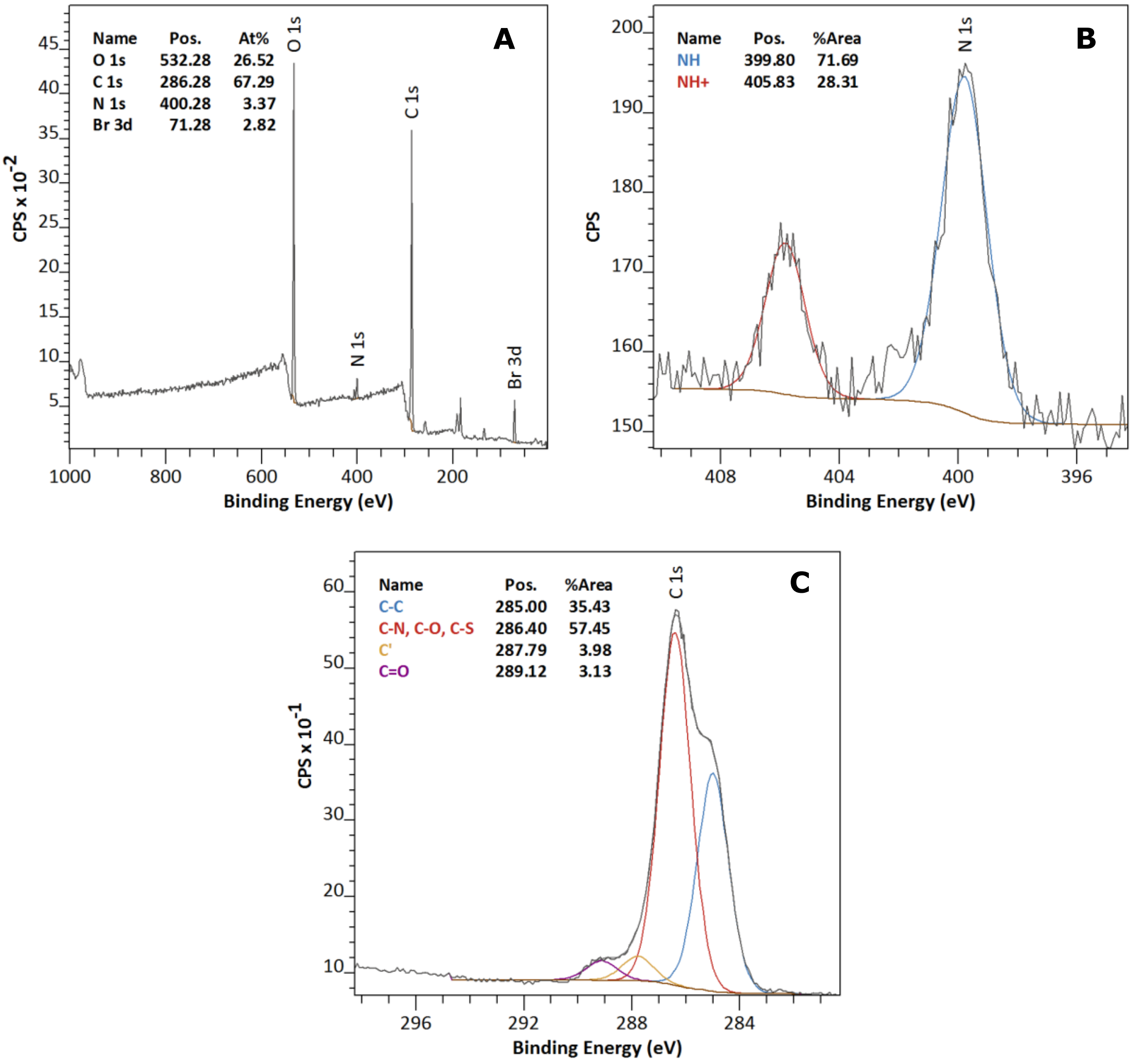

Figure S2. XPS spectra of Dynabeads functionalized with α-bromoisobutyryl bromide: (A) wide-scan and narrow-scans of (B) N 1s and (C) C 1s. The peak labelled as C' originates from the bare Dynabead (see Figure S1). Most indicative for the introduction of the α-bromoisobutyryl bromide is the appearance of a distinct Br 3d signal at 71.28 eV in the wide-scan in (A).

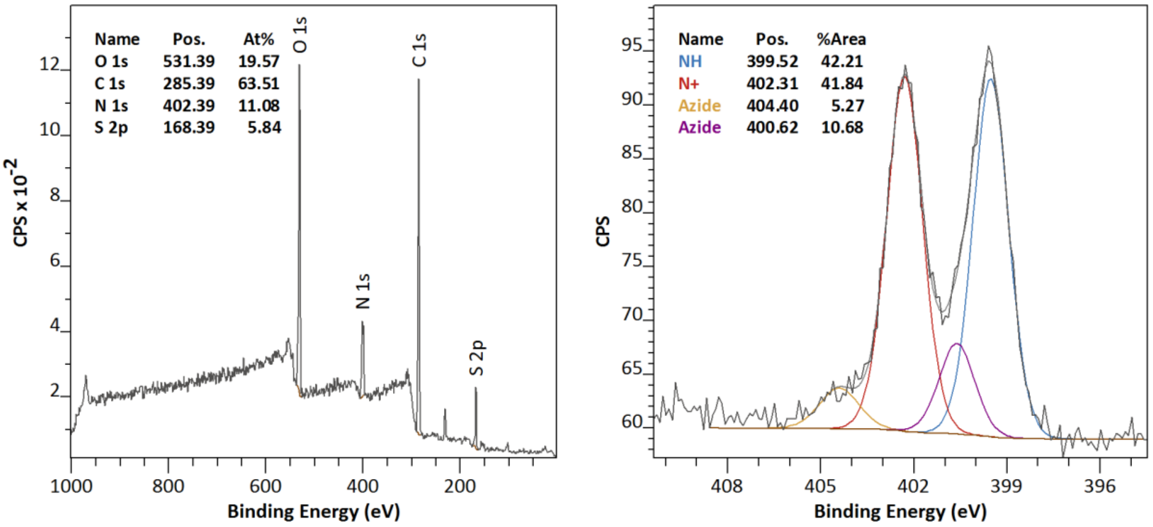

Figure S3. XPS wide-scan (left) and XPS N 1s narrow-scan (right) spectrum of *p*SB-*co*-(azido)$_{16\%}$ beads. The spectra show the successful growth of zwitterionic *co*-polymer brushes of SB and azido-SB monomer from beads as seen by the appearance of sulfur signals in the wide-scans, the 1:1 ratio of ammonium versus amide nitrogen peak and the appearance of N 1s azide peaks.

2. DYNAMIC LIGHT SCATTERING DATA

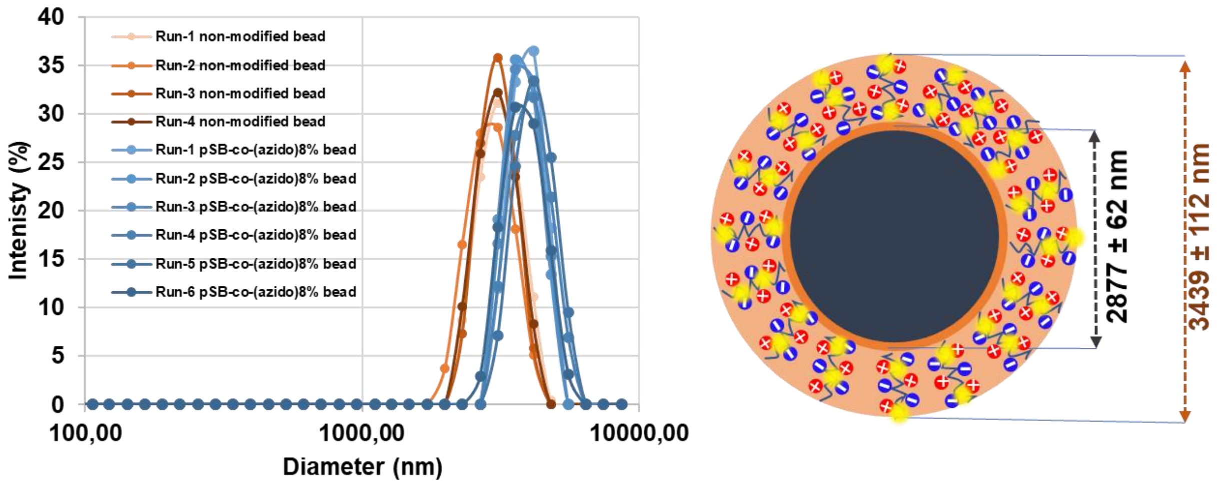

	Mean d.nm
Run 1 – non-modified beads	2935
Run 2 – non-modified beads	2789
Run 3 – non-modified beads	2889
Run 4 – non-modified beads	2893
Mean	**2877**
Standard deviation	62
Run 1 – *p*SB-*co*-(azido)8% beads	3337
Run 2 – *p*SB-*co*-(azido)8% beads	3360
Run 3 – *p*SB-*co*-(azido)8% beads	3389
Run 4 – *p*SB-*co*-(azido)8% beads	3532
Run 5 – *p*SB-*co*-(azido)8% beads	3619
Run 6 – *p*SB-*co*-(azido)8% beads	3394
Mean	**3439**
Standard deviation	112

Figure S4. Hydrodynamic diameter of non-modified and *p*SB-*co*-(azido)$_{8\%}$ beads analyzed by Dynamic Light Scattering (DLS). Beads were suspended in water. Data presented as graph, in a table and as schematic representation.

3. FULL SIZE SDS-PAGE GELS

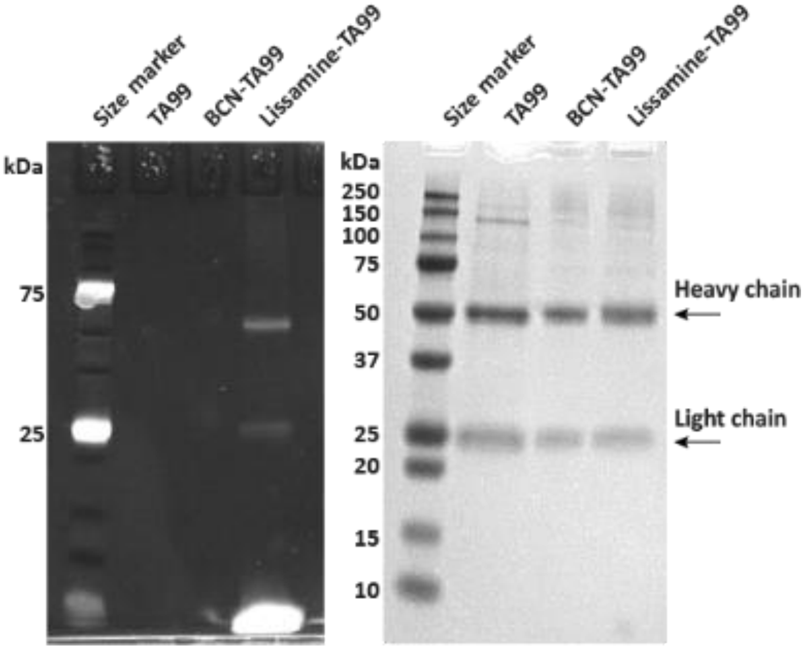

Figure S5. Full size SDS-PAGE gel of untreated TA99 antibody, TA99 labeled with BCN-NHS (BCN-TA99), and BCN-TA99 reacted with Azide-Lissamine (Liss-TA99). Visualization by fluorescence (left) using Bio-Rad ChemiDoc™ XRS+ with standard 580 AF 120 filter followed by Coomassie blue staining (right). The very bright band at low molecular weight corresponds to free Azide-Lissamine.

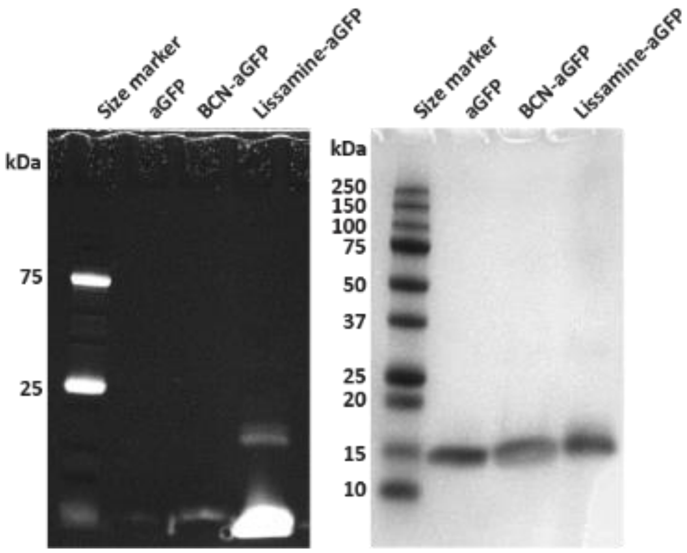

Figure S6. Full size SDS-PAGE gel of untreated aGFP antibody, aGFP labeled with BCN-NHS (BCN-aGFP), and BCN-aGFP reacted with Azide-Lissamine (Lissamine-aGFP). Visualization by fluorescence (left) using Bio-Rad ChemiDoc™ XRS+ with standard 580 AF 120 filter followed by Coomassie blue staining (right). The aGFP antibody used is different from a standard IgG mouse antibody (hence no bands of heavy and light chains), it is the 13.9 kDa VHH domain of a 27 kDa single chain camelid antibody. The very bright band at low molecular weight corresponds to free Azide-Lissamine.

4. ADDITIONAL FLOW CYTOMETRY DATA

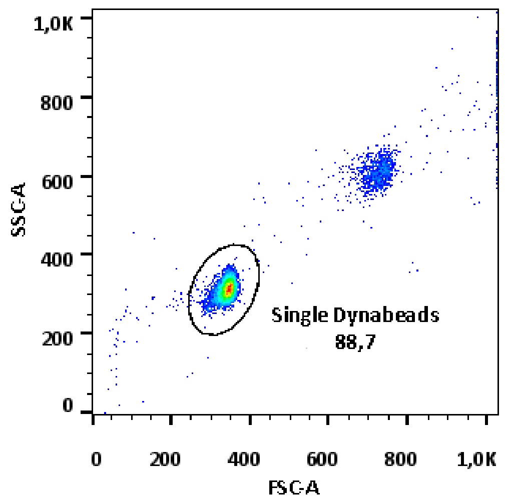

Figure S7. Typical side/forward scatter plot of Dynabeads as obtained by flow cytometry. A total of 10,000 single beads were acquired per sample and used in further analysis. This type of gating was used for Figures 2-3 and S9.

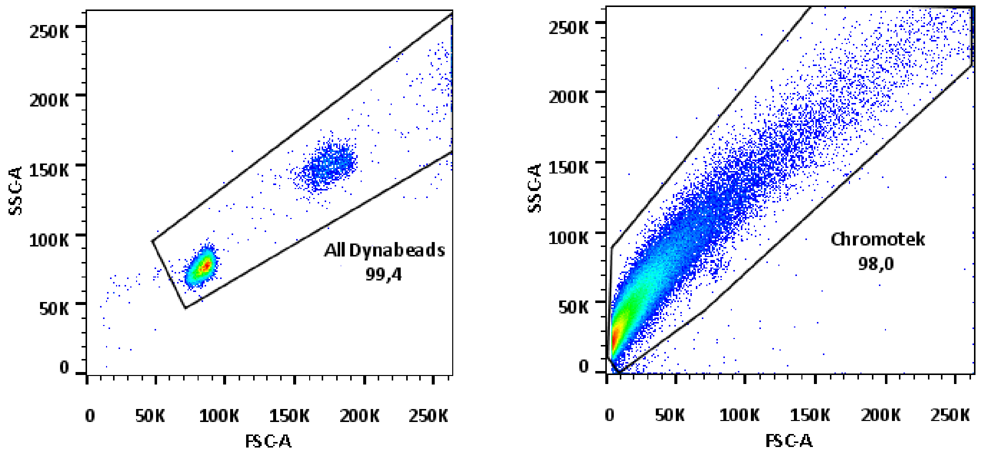

Figure S8. Example of flow cytometry gating for the comparison of Dynabeads and Chromotek beads. All bead events are selected, 10,000 bead events were measured per sample. This type of gating was used for Figures 4. SSC = side scatter, FSC = forward scatter.

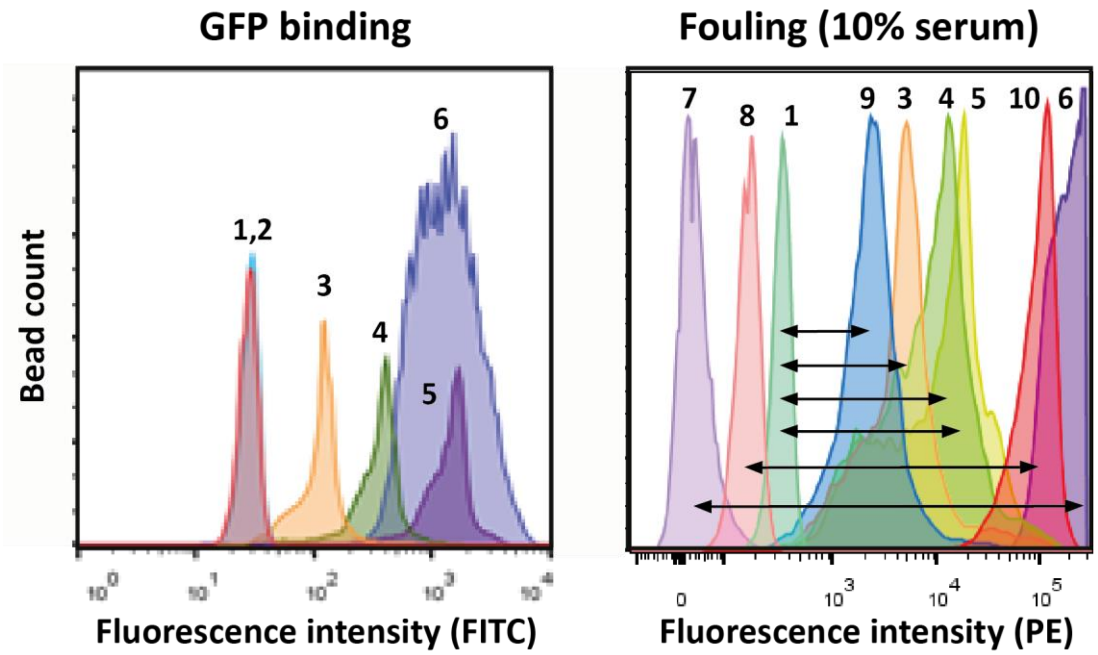

1. *p*SB-*co*-(azido)$_{8\%}$ + PBS
2. *p*SB-*co*-(azido)$_{8\%}$ + PBS + GFP
3. *p*SB-*co*-(aGFP)$_{8\%}$ + PBS + GFP + Serum-biotin + Strep-PE (0.25 mg/mL aGFP)
4. *p*SB-*co*-(aGFP)$_{8\%}$ + PBS + GFP + Serum-biotin + Strep-PE (1 mg/mL aGFP)
5. *p*SB-*co*-(aGFP)$_{8\%}$ + PBS + GFP + Serum-biotin + Strep-PE (4 mg/mL aGFP)
6. Chromotek + PBS + GFP + Serum-biotin + Strep-PE
7. Chromotek + PBS
8. NM + PBS
9. *p*SB-*co*-(azido)$_{8\%}$ + PBS + Serum-biotin + Strep-PE
10. NM + PBS + Serum-biotin + Strep-PE

Figure S9. Flow cytometry histogram plots showing fouling and GFP capture by aGFP Chromotek beads, non-modified Dynabeads (NM), *p*SB-*co*-(azido)$_{8\%}$ and *p*SB-*co*-(aGFP)$_{8\%}$ beads prepared with an aGFP antibody concentration of 0.25, 1 or 4 mg/mL. All beads were incubated in 10% biotinylated serum spiked with GFP (10 µg/mL), and after washing incubated with Strep-PE to stain for fouling by biotinylated serum proteins.

5. IP-MS

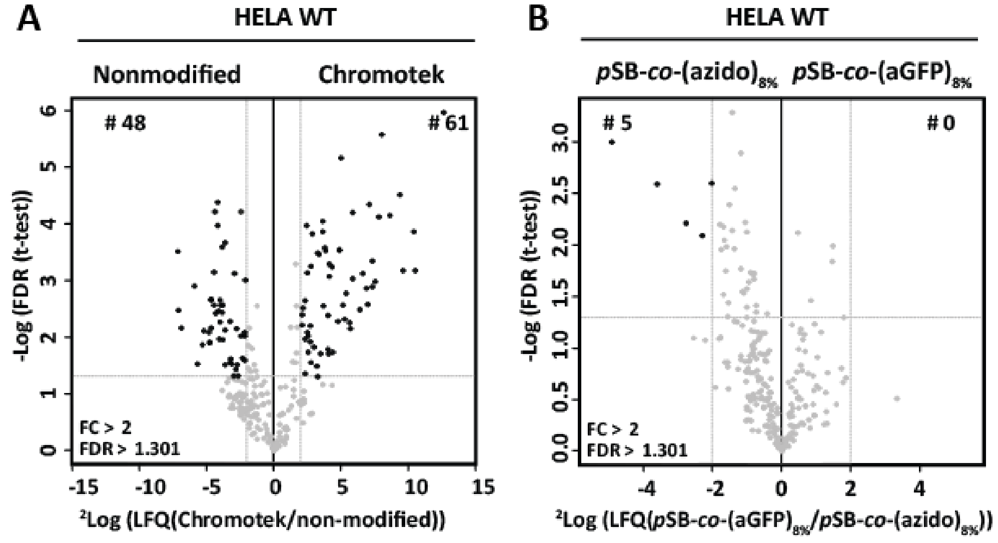

Figure S10. Mass spectrometry-based analysis of protein enrichment using non-modified Dynabeads, *p*SB-*co*-(azido)$_{8\%}$, *p*SB-*co*-(aGFP)$_{8\%}$ and Chromotek beads represented as volcano plots. Statistically enriched proteins are identified using an FDR-corrected t-test. The relative label-free quantification (LFQ) intensities (*x*-axis) are plotted against the $-^{10}$log-transformed *P*-values of the t-test (*y*-axis). A) Non-modified and Chromotek beads, and B) *p*SB-*co*-(azido)$_{8\%}$ and *p*SB-*co*-(aGFP)$_{8\%}$ beads, subjected to WT HeLa whole-cell lysate. These data illustrate that Chromotek beads show higher non-specific binding of proteins than non-modified Dynabeads (A), and that antifouling beads with unmodified azido groups (*p*SB-*co*-(azido)$_{8\%}$) have slightly higher non-specific protein binding in comparison to antifouling beads to which the aGFP antibody is attached (*p*SB-*co*-(aGFP)$_{8\%}$ beads) (B).

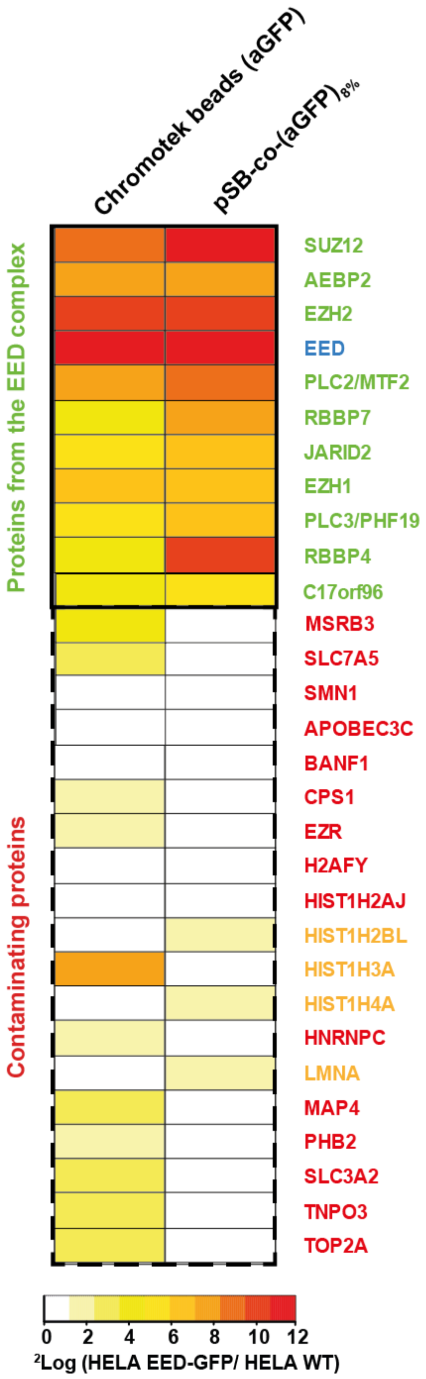

Figure S11. Heat-map of immunoprecipitation experiment targeting the EED-GFP fusion protein with Chromotek beads and *p*SB-*co*-$(aGFP)_{8\%}$ beads. Identified proteins are listed and color-coded (white indicates that the specified protein was not found at a concentration different from the control WT Hela cell lysate, red indicates highly enriched as compared to the control samples). Identified proteins shown in the solid line block (the protein names in green) are known components of the EED complex. Protein names in red indicate possible contaminating proteins, while protein names in yellow might represent possible interactors with the EED/PRC2 complex (see also main text).
